# Identification of four genes and biological characteristics of esophageal squamous cell carcinoma by integrated bioinformatics analysis

**DOI:** 10.1186/s12935-021-01814-1

**Published:** 2021-02-18

**Authors:** Yexun Song, Xianyao Wang, Fengjun Wang, Xiaowei Peng, Peiyu Li, Shaojun Liu, Decai Zhang

**Affiliations:** 1 Department of Otolaryngology-Head Neck Surgery, The Third Xiangya Hospital of Central South University, Changsha, 410013 Hunan China; 2 Department of Otolaryngology-Head Neck Surgery, The Xiangya Hospital of Central South University, Changsha, 410008 Hunan China; 3 Department of Clinical Laboratory, The Third Xiangya Hospital of Central South University, Changsha, 410013 Hunan China; 4 Department of Oncology Plastic Surgery, Hunan Province Cancer Hospital, Changsha, 410007 Hunan China; 5 Department of Gastroenterology, The Third Xiangya Hospital of Central South University, Changsha, 410013 Hunan China; 6 Key Laboratory of Nonresolving Inflammation and Cancer, Changsha, 410013 Hunan China

**Keywords:** Esophageal squamous cell carcinoma, Bioinformatic analysis, Differentially expressed genes, Hub genes, Biomarker

## Abstract

**Background:**

Esophageal squamous cell carcinoma (ESCC) has become one of the most serious diseases affecting populations worldwide and is the primary subtype of esophageal cancer (EC). However, the molecular mechanisms governing the development of ESCC have not been fully elucidated.

**Methods:**

The robust rank aggregation method was performed to identify the differentially expressed genes (DEGs) in six datasets (GSE17351, GSE20347, GSE23400, GSE26886, GSE38129 and GSE77861) from the Gene Expression Omnibus (GEO). The Search Tool for the Retrieval of Interacting Genes (STRING) database was utilized to extract four hub genes from the protein–protein interaction (PPI) network. Module analysis and disease free survival analysis of the four hub genes were performed by Cytoscape and GEPIA. The expression of hub genes was analyzed by GEPIA and the Oncomine database and verified by real-time quantitative PCR (qRT-PCR).

**Results:**

In total, 720 DEGs were identified in the present study; these genes consisted of 302 upregulated genes and 418 downregulated genes that were significantly enriched in the cellular component of the extracellular matrix part followed by the biological process of the cell cycle phase and nuclear division. The primary enriched pathways were hsa04110:Cell cycle and hsa03030:DNA replication. Four hub genes were screened out, namely, SPP1, MMP12, COL10A1 and COL5A2. These hub genes all exhibited notably increased expression in ESCC samples compared with normal samples, and ESCC patients with upregulation of all four hub genes exhibited worse disease free survival.

**Conclusions:**

SPP1, MMP12, COL10A1 and COL5A2 may participate in the tumorigenesis of ESCC and demonstrate the potential to serve as molecular biomarkers in the early diagnosis of ESCC. This study may help to elucidate the molecular mechanisms governing ESCC and facilitate the selection of targets for early treatment and diagnosis.

## Background

Esophageal cancer (EC), which is one of the most common malignant diseases, has become the sixth leading cause of cancer deaths worldwide [1]. Esophageal squamous cell carcinoma (ESCC) is one of the primary histological subtypes of EC, accounting for ~ 90 % of EC cases in China [2–4]. Although notable advances have been made in diagnostic and multidisciplinary therapies for ESCC, the 5-year survival rate for ESCC remains below 20 %. Many studies have demonstrated that the lack of specific biomarkers for ESCC represents one of the key factors contributing to the low survival rate [5–8]. Although there are extensive studies on the mechanisms governing ESCC formation and progression, the causes of ESCC have not been elucidated to date. Therefore, identifying the hub genes associated with ESCC is critical to determine the molecular mechanisms governing ESCC and to select ESCC therapeutic candidate targets.

As a high-throughput technology, microarray technology has been applied to molecular biomarkers and key factor exploration in various cancers [9–11]. Furthermore, the Gene Expression Omnibus (GEO) database and The Cancer Genome Atlas (TCGA) database are increasingly recognized by researchers, and an increasing number of tumor-associated genes have been investigated through bioinformatic analysis [12, 13]. Moreover, using systematic analysis of gene expression can rapidly filter DEGs that may have important effects on cancer progression [14]. The data from these public databases may help to characterize the development and molecular mechanism of ESCC after reanalysis. To date, various gene chips have been utilized in many studies to identify key molecular factors for ESCC, and various genes, mRNAs and miRNAs have been detected [15–17]. For example, 280 DEGs that consisted of 96 upregulated DEGs and 184 downregulated DEGs and 26 differentially expressed miRNAs were found by miRNA-mRNA integrated analysis of the data from the GEO and TCGA databases by Zhang [18]; also, Yang et al. identified several hub genes and therapeutic drugs in ESCC via an integrated bioinformatics strategy [19]. However, the existence of tumor heterogeneity may lead to inconsistent and variable results. To date, few reliable biomarkers have been identified and utilized for ESCC. In addition, although many genes have been determined to be involved in ESCC, the mechanisms underlying the involvement of these genes in the development of ESCC have not been elucidated. Therefore, it is urgently important to identify effective molecular biomarkers that will be crucial to the diagnosis and treatment of ESCC patients, and the hub genes in ESCC along with the biological pathways associated with the DEGs are investigated in the present study.

In this study, the expression profiles of mRNAs were collected in normal and ESCC tissues from the Gene Expression Omnibus (GEO) and The Cancer Genome Atlas (TCGA) databases, and DEGs were identified by the Robust Rank Aggreg package in R. Furthermore, Gene Ontology (GO) annotation and Kyoto Encyclopedia of Genes and Genomes (KEGG) pathway analyses were performed to assess the functional pathways of DEGs, and hub genes were extracted from a protein–protein interaction (PPI) network. Moreover, to better understand the function of these hub genes in ESCC, GEPIA database was employed to evaluate the disease free survival of the four hub genes, and the expression of these genes was also analyzed using the GEPIA and Oncomine databases and real-time quantitative PCR (qRT-PCR).

## Materials and methods

### Collection of tissue specimens

10 ESCC and 10 esophageal normal tissues specimens were obtained from patients in the Third Xiangya Hospital (Changsha, People’s Republic of China). All patients were informed of the investigational nature of the study. Written informed consent was obtained from them before the experiment. This study was reviewed and approved by the Ethics Committee of the Third Xiangya Hospital. All tissue samples were indentified by histopathological evaluation, and stored at liquid nitrogen until used.

### Data acquisition and preprocessing

Six datasets (GSE17351, GSE20347, GSE23400, GSE26886, GSE38129 and GSE77861) were downloaded from Gene Expression Omnibus (GEO, https://www.ncbi.nlm.nih.gov/geo/) used GEO query described by Sean and Meltzer [20]. The detailed information of all the six GEO datasets with gene expression profiles in ESCC and normal tissues was listed in Table 1. The raw microarray data of expression files were normalized and log2-transformed. DEGs were identified by the Bioconductor Limma package and then robust rank aggregation method was used to integrate and ranke all of the DEGs from six GEO datasets. In addition, the edgeR package was used to screen DEGs with thresholds of |log2fold change|>1 and the thresholds of the adjusted *p*-value(FDR) < 0.05.

**Table 1:** The detailed information of the six GEO datasets

Dataset	Numbers of samples (Tumor / Normal)	Array types	Experiment type	Origin
Nakagawa et al. GSE17351	5/5	Affymetrix Human Genome U133 Plus 2.0 Array	mRNA	Carcinogenesis
Clifford et al. GSE20347	17/17	Affymetrix Human Genome U133 Plus 2.0 Array	mRNA	BMC Genomics
Su et al. GSE23400	53/53	Affymetrix Human Genome U133B Array	mRNA	Int J Epidemiol
Wang et al. GSE26886	9/19	Affymetrix Human Genome U133 Plus 2.0 Array	mRNA	BMC Cancer
Hu et al. GSE38129	30/30	Affymetrix Human Genome U133A Array	mRNA	BMC Genomics
Erkizan et al. GSE77861	7/7	Affymetrix Human Gene Expression Array	mRNA	BMC Cancer

*GEO* Gene Expression Omnibus

### Gene ontology (GO) analysis and Kyoto encyclopedia of Genes and Genomes (KEGG) pathway analysis

The DEGs from GEO database were analyzed by an online program Database for annotation, visualization and integrated discovery (DAVID) (http://david.abcc.ncifcrf.gov/) [21]. The GOchord R package and DAVID database were used to perform GO (Gene Ontology) analysis and KEGG pathway maps with cut-off *p* < 0.05, respectively [22].

### Protein–protein interaction (PPI) network construction

According to the DEGs identified, protein–protein interaction network was performed by the Search Tool for the Retrieval of Interacting Genes (STRING) (https://string-db.org/) with the threshold = 0.9.The hub genes were identified by Cytoscape and modules of hub genes from the PPI network was screened by the Molecular Complex Detection (MCODE) with the following default parameters: node score cut-off = 0.2, cut-off = 2, k-core = 2, and max depth = 100 [23].

### Hub genes analysis

The seed genes in modules with the most connectivities referred to hub genes and TCGA KIRC data was used to perform validation using GEPIA database [24]. The RNA-sequencing (RNA-seq) data for hub genes were downloaded from the The Cancer Genome Atlas (TCGA, https://tcga-data.nci.nih.gov/tcga/) database. The analysis for expression level of hub genes between normal esophageal samples (n = 182) and ESCC samples (n = 286) was based on GTEx data in GEPIA from TCGA. Oncomine database was used to further analyse the expression level of hub genes with clinical traits [25, 26]. Logrank value *p* < 0.05 was considered to be statistically significant.

### Total RNA isolation and real‐time quantitative PCR (qRT-PCR)

Total RNA from normal esophageal samples (n = 10) and ESCC samples (n = 10) were isolated using RNeasy Mini Kit (Cat.74101, Qiagen, Germany) according to the manufacturer’s instruction. The synthesis of cDNA used for genes were finished using the BestarTM qPCR RT kit (DBI; #DBI-0) from 2µg RNA. The relative mRNA levels of SPP1, MMP12, COL10A1 and COL5A2 were determined by qRT-PCR method using a 20µL reaction system. The PCR process was done on an ABI PRISM 7500 real-time PCR system (Applied Biosystems, Carlsbad, CA, USA) using the following settings: 95℃ for 2 min, followed by 40 cycle of 94℃ for 20 S, 58℃ for 20 S and 72℃ for 20s. GAPDH was used as the internal normalized reference to genes. The fold change was determined via 2 − ΔΔCt (ΔΔCt = (ΔCt of genes of interest) − (ΔCt of GAPDH). The primer sequences used as follows: SPP1: F: 5’-TTTGTTGTAAAGCTGCTTTTCCTC-3’R: 5’-GAATTGCAGTGATTTGCTTTTGC-3’; MMP12: F: 5’-ACGTGGCATTCAGTCCCTGT-3’R: 5’-AACACTGGTCTTTGGTCTCTCAGAA-3’; COL10A1: F: 5’-ATGCTGCCACAAATACCCTTT-3’R: 5’-GGTAGTGGGCCTTTTATGCCT-3’; COL5A2: F: 5’-GGAAGAAGACGAGGATGAAGGATA-3’; R: 5’-CAGGAC CAGAAGGACCAACT-3’.

### Statistical analysis

All statistical analysis in present study were calculated using SPSS 19.0 (SPSS Inc., Chicago, IL, USA). All of the data were presented as mean ± standard deviation (SD). Statistical significance between two groups was evaluated by Student’s t test between two groups. *p* < 0.05 was statistically significant. All experiments were repeated at least three times.

## Results

### Identification of DEGs among six GEO datasets

**Fig. 1:**
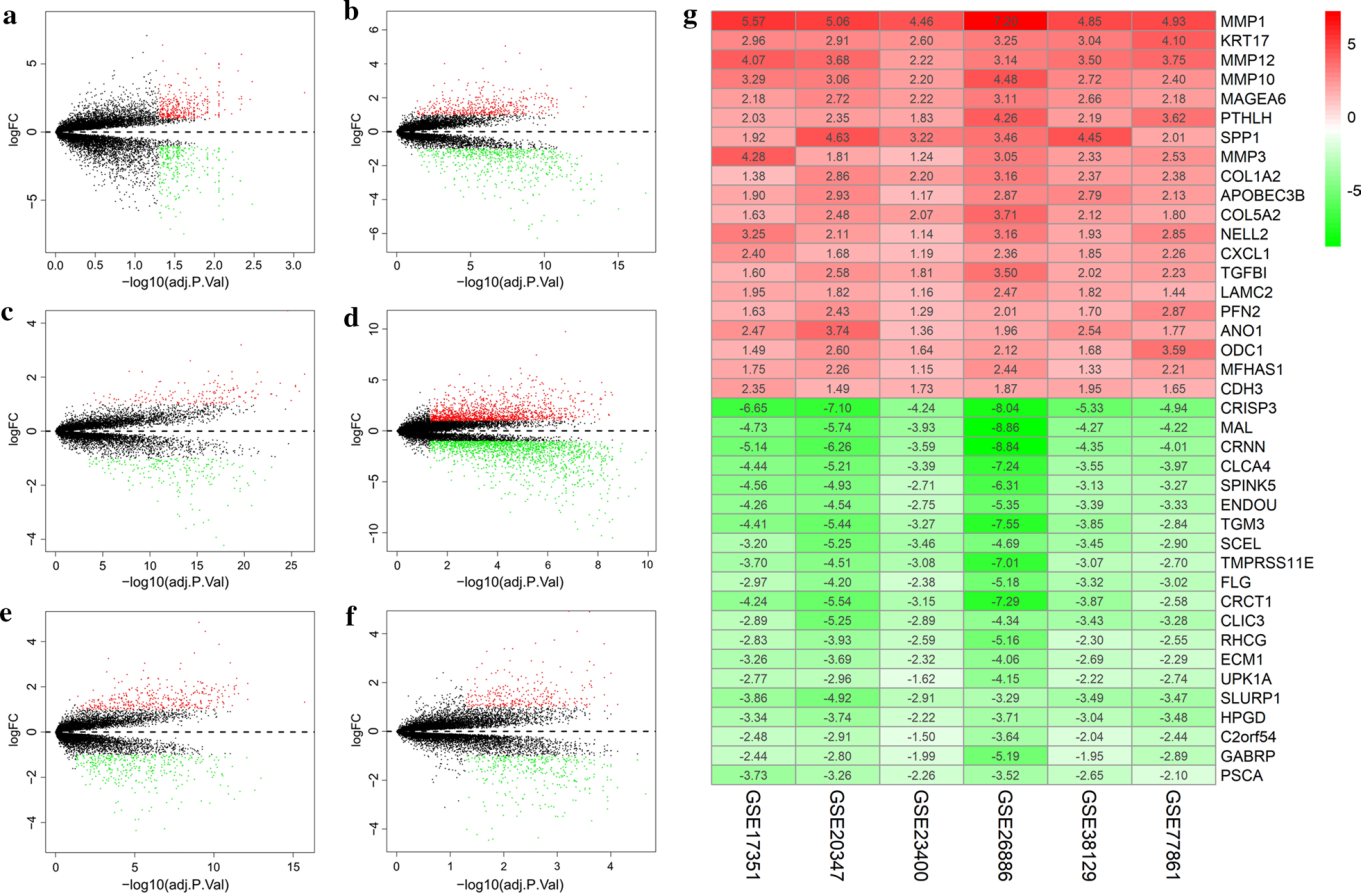
Identification of DEGs among each GEO data set. **a**–**f** Volcano plots of the distribution of DEGs in each data set. Red dots on the top indicate upregulated genes, green dots on the bottom indicate downregulation, and black dots indicate genes with no statistically significant difference. **g** The expression heat map of the 20 robust DEGs by using the RRA method

Six datasets with a total of 131 normal samples and 121 ESCC samples were downloaded using the GEO by getGEO function in the GEOquery package; the datasets were GSE17351, GSE20347, GSE23400, GSE26886, GSE38129 and GSE77861 (Table 1). In total, 302 upregulated DEGs and 418 downregulated DEGs were identified in the GSE17351, GSE20347, GSE23400, GSE26886, GSE38129 and GSE77861 datasets (Fig. 1a–f). Specifically, after reprocessing was performed on the raw microarray data of the expression files, 720 DEGs were screened out; 302 of the DEGs were upregulated, and 418 were downregulated (Additional file 1: Table S1). The top 20 significantly differentially upregulated and downregulated genes are listed in Fig. 1g.

### GO and KEGG analysis

**Fig. 2:**
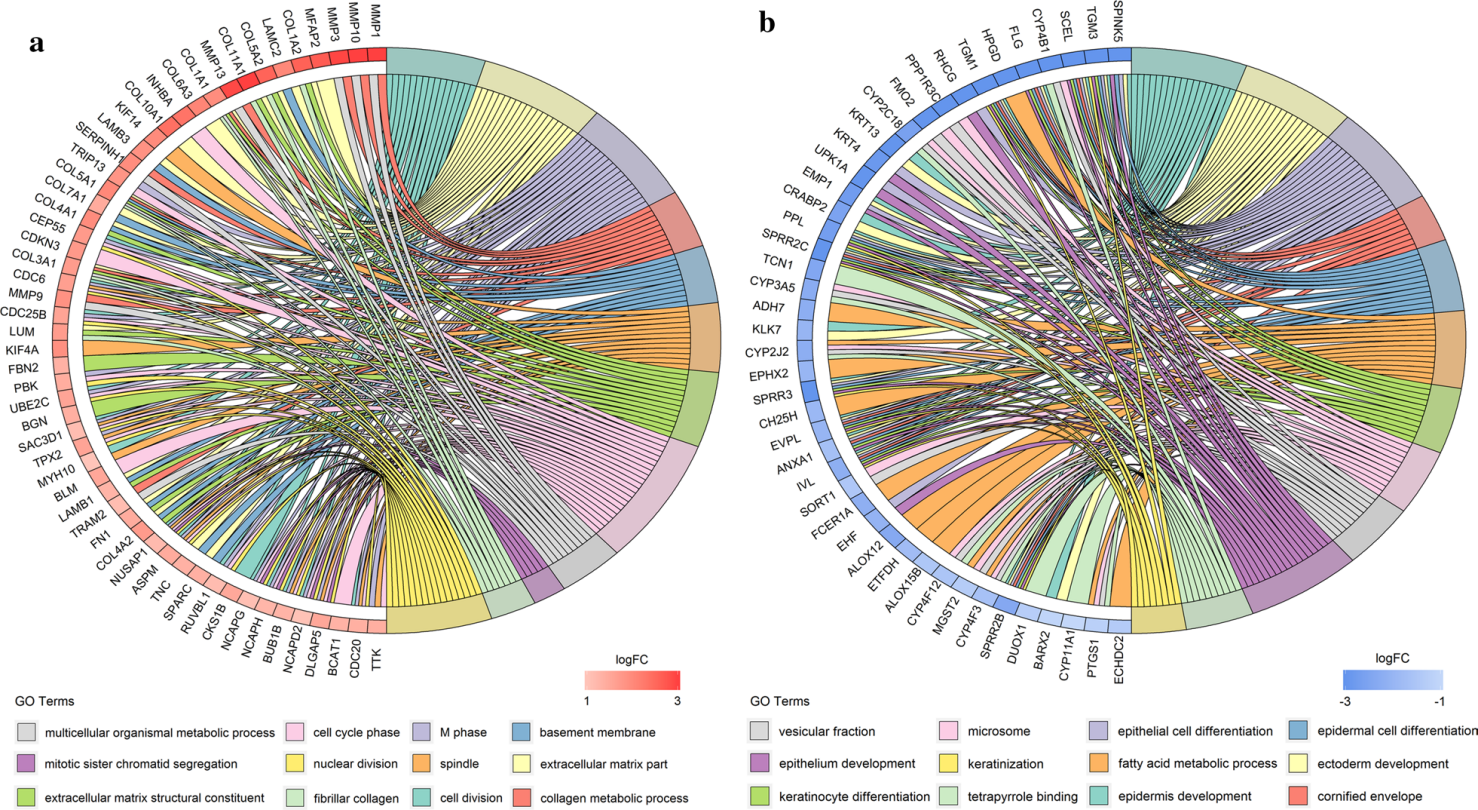
GO enrichment analyses of the up- and downregulated DEGs. **a** GO enrichment of the upregulated DEGs; **b** GO enrichment of the downregulated DEGs

**Fig. 3:**
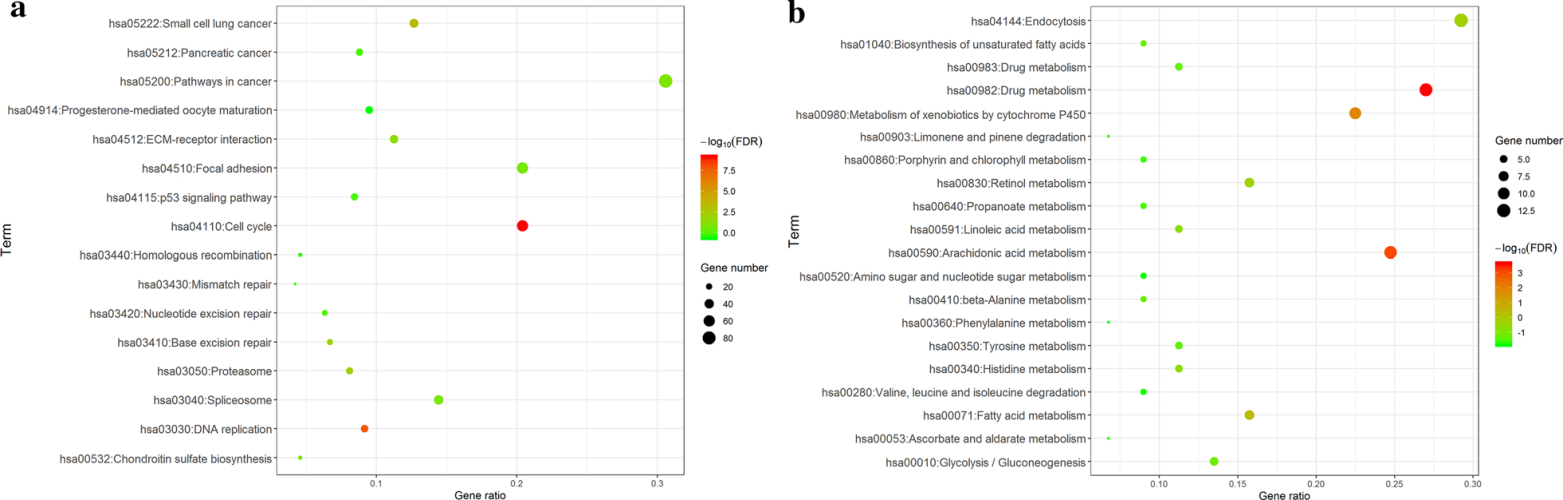
Bubble map of KEGG pathway analysis. **a** Bubble map of KEGG pathway analysis for upregulated DEGs. **b** Bubble map of KEGG pathway analysis for downregulated DEGs. The horizontal axis represents the fold enrichment of pathways, and the vertical axis represents pathway names. The size of bubbles represents the number of genes, and the shade of color depends on the *p*-value

To determine the function of DEGs in ESCC, the up- and downregulated DEGs were subjected to GO analysis by the GOChord R package. The GO categories of molecular function (MF), biological process (BP) and cellular component (CC) for DEGs were significantly enriched, and the top 12 GO terms of the DEGs with upregulation and downregulation are listed in Additional file 2: Table S2. Based on the GOChord plotting function, for BP, the upregulated DEGs were significantly enriched in response to cell cycle phase (GO:0022403), nuclear division (GO:0000280), M phase (GO:0000279), cell division (GO:0051301), collagen metabolic process (GO:0032963), the multicellular organismal metabolic process (GO:0044236) and mitotic sister chromatid segregation (GO:0000070), and the downregulatedDEGs were significantly enriched in epidermis development (GO:0008544), ectoderm development (GO:0007398), epithelial cell differentiation (GO:0030855), epidermal cell differentiation (GO:0009913), the fatty acid metabolic process (GO:0006631), keratinocyte differentiation (GO:0030216), epithelium development (GO:0060429) and keratinization (GO:0031424) (Fig. 2a and b). Regarding MF, the upregulated DEGs were significantly enriched in extracellular matrix structural constituents (GO:0005201), and the downregulated DEGswere significantly enriched in tetrapyrrole binding (GO:0046906) (Fig. 2a and b). Concerning CC, the upregulated DEGs were significantly enriched in extracellular matrix component (GO:0044420), spindle (GO:0005819), fibrillar collagen (GO:0005583), and basement membrane (GO:0005604), and the downregulated DEGs were significantly enriched in cornified envelope (GO:0001533), microsome (GO:0005792) and vesicular fraction (GO:0042598) (Fig. 2a and b). These results of GO analysis identified the functions of the DEGs in ESCC development and progression. KEGG pathway analysis was used for further analysis of all DEGs. The upregulated genes were significantly enriched in hsa04110: cell cycle, hsa03030:DNA replication, hsa05222: small-cell lung cancer, hsa03050: proteasome and hsa03410: base excision repair (Fig. 3a and (Additional file 3: Table S3), and the downregulated DEGs were most significantly enriched in hsa00982: drug metabolism, hsa00590: arachidonic acid metabolism and hsa00980: metabolism of xenobiotics by cytochrome P450 (Fig. 3b and Additional file 2: Table S2).

### Construction of PPI network and module analysis

**Fig. 4:**
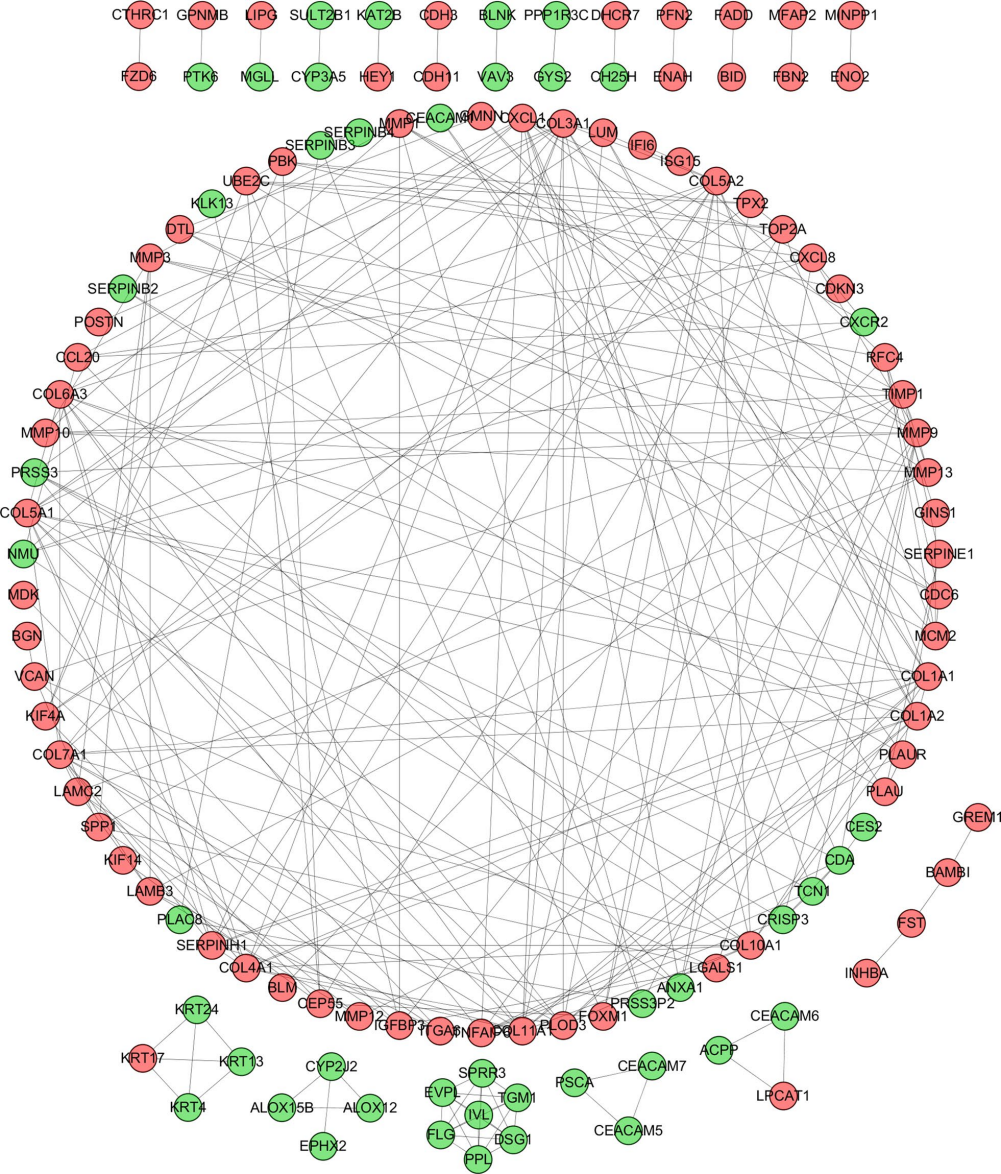
Protein–protein interaction network of DEGs using STRING. Color and size represent the connectivity degree of nodes; network nodes stand for proteins (represented with gene names); the color in each node corresponds to the expression of DEGs in comparison to normal esophageal samples, red for upregulation and green for downregulation. The nodes represent the proteins expressed by DEGs, and the edges between two nodes indicate the physical interactions

To understand the molecular mechanisms that govern ESCC progression, a PPI network was constructed using the STRING database with the threshold = 0.9, and all the nodes without connections were removed from the PPI network. Subsequently, the PPI network was analyzed, and the most highly connected clusters were extracted by the MCODE plug-in in Cytoscape. Genes in this cluster, namely, SPP1, MMP12, COL10A1 and COL5A2, were at the core of the whole network (Fig. 4). Therefore, these four genes were considered to be hub genes and utilized for further analysis. These genes were all significantly upregulated in ESCC samples compared with normal samples.

### Hub gene analysis

**Fig. 5:**
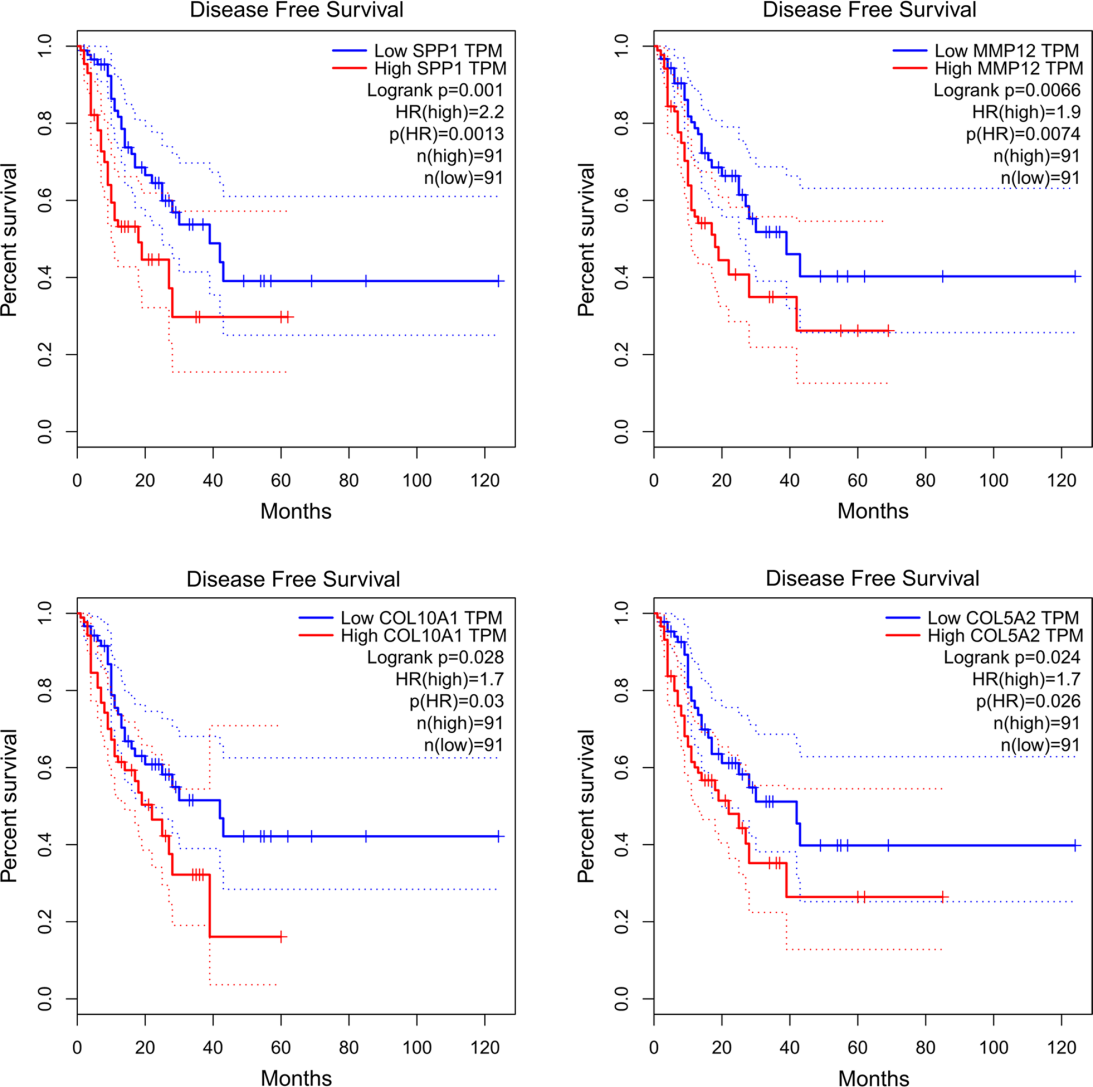
Survival analysis of four hub genes in ESCC based on TCGA and GTEx data in GEPIA. Disease-free survival analyses of hub genes were performed using GEPIA database. Logrank *p* < 0.05 was considered to be significant

**Fig. 6:**
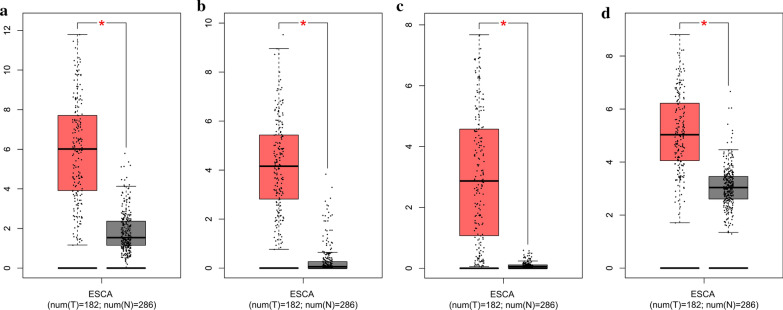
Validation of the expression levels of the four hub genes between normal esophageal samples and ESCC samples based on TCGA and GTEx data in GEPIA. **a**–**d**, Expression levels of SPP1, MMP12, COL10A1 and COL5A2 in normal esophageal samples and ESCC samples. All of the data are presented as means ± SD. Significant differences were defined by a *p*-value < 0.05

**Fig. 7:**
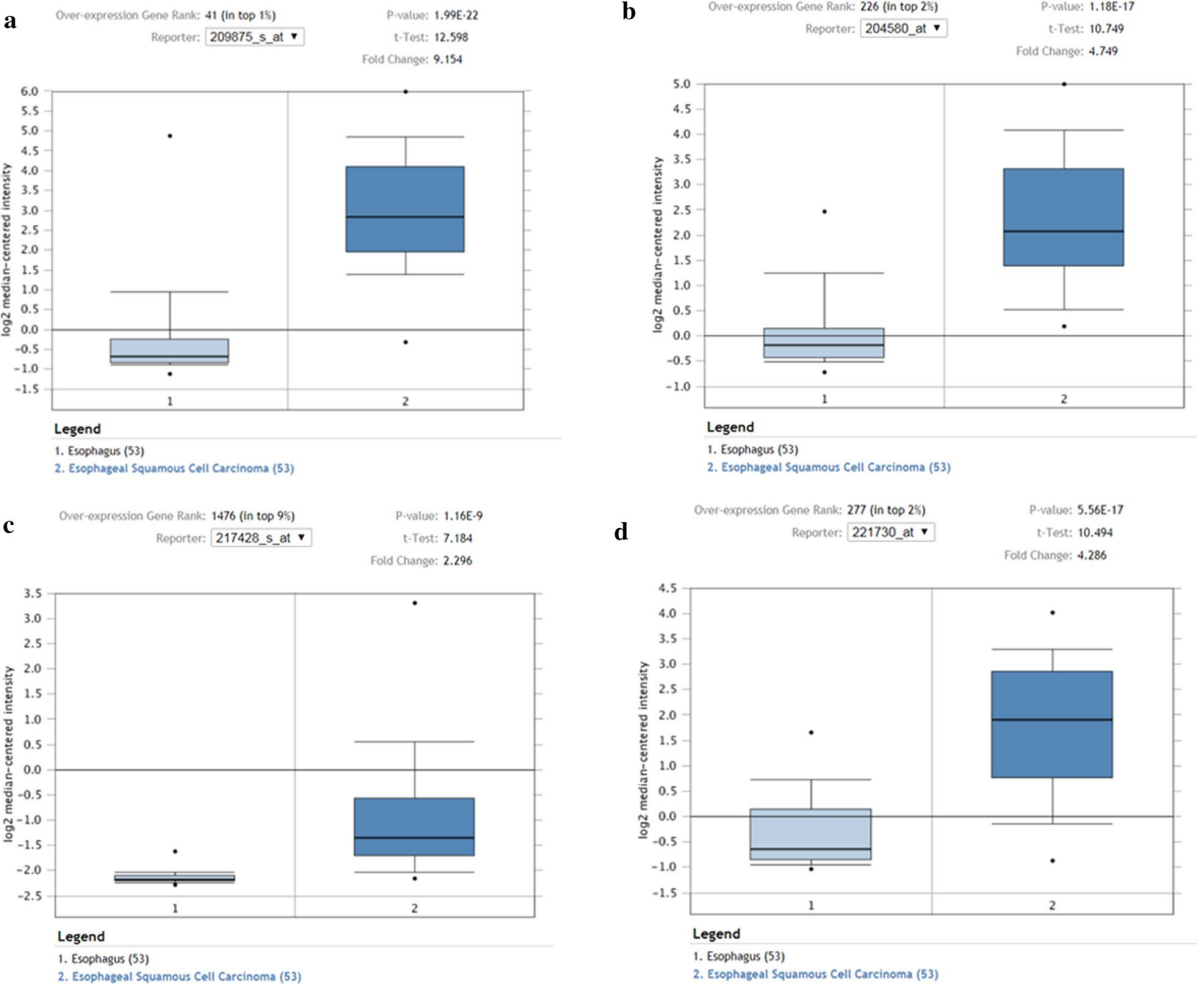
Validation of the expression levels of the four hub genes between normal esophageal samples and ESCC samples based on Oncomine data. **a**–**d**, Expression levels of SPP1, MMP12, COL10A1 and COL5A2 in normal esophageal samples and ESCC samples

To determine the survival of SPP1, MMP12, COL10A1 and COL5A2, hub genes were analyzed using GEPIA database. As shown in Fig. 5, ESCC patients with upregulation of all four hub genes showed worse disease free survival. Subsequently, the expression status of hub genes was further validated using the GEPIA and Oncomine databases. As shown in Fig. 6a–d and 286 normal esophageal samples and 182 ESCC samples were identified in the GEPIA and GTEx databases based on TCGA. The expression levels of all four hub genes were significantly increased in ESCC samples compared with normal samples (*p* < 0.05). These results were also confirmed by the expression changes in the Oncomine database for SPP1(*p* = 1.99E−22), MMP12 (*p* = 1.18E−17), COL10A1 (*p* = 1.16E−9) and COL5A2 (*p* = 5.56E−17) (Fig. 7a–d).

### Expression validation of the four hub genes by qRT-PCR

**Fig. 8:**
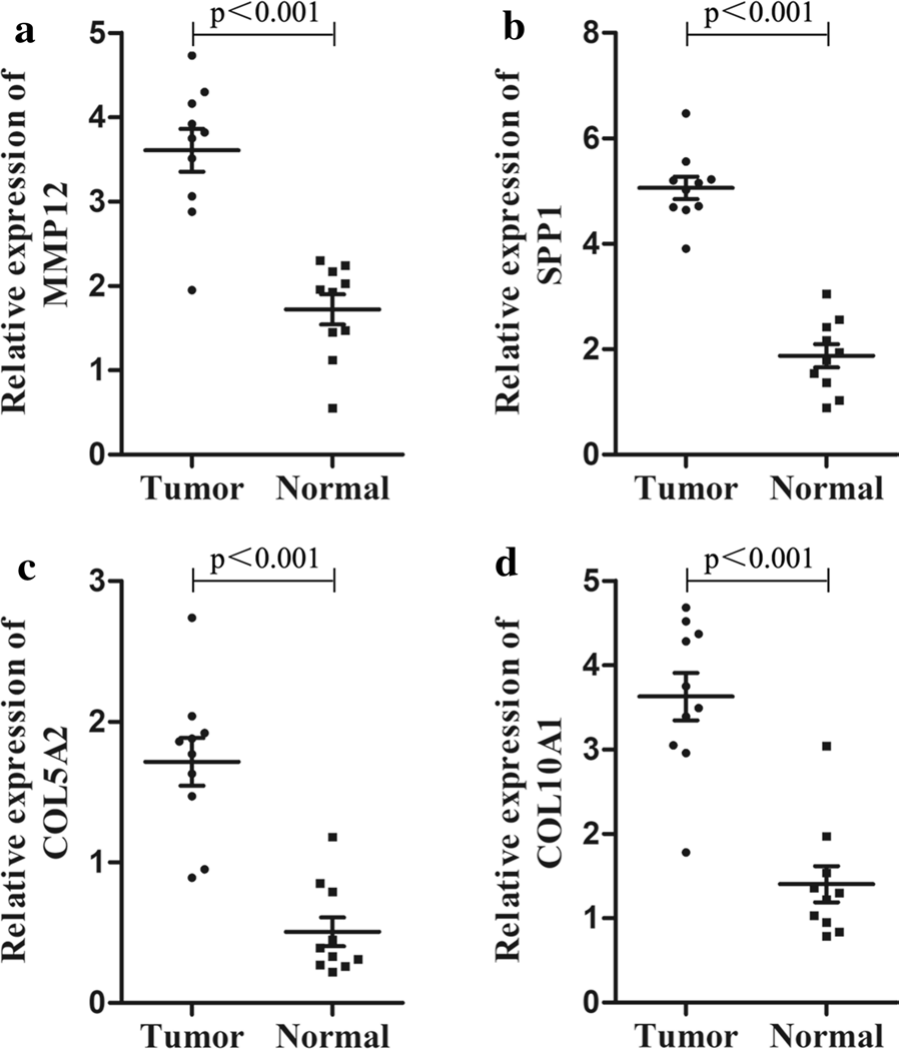
Validation of the expression levels of the four hub genes between normal esophageal samples (n = 10) and ESCC samples (n = 10) by PCR analysis. All of the data are presented as means ± SD. Significant differences were defined by a *p*-value < 0.001

To better characterize the expression levels of the four hub genes in normal and ESCC tissues, 10 normal esophageal samples and 10 ESCC samples were collected. As shown in Fig. 8, compared with normal esophageal samples, the expression levels of SPP1, MMP12, COL10A1 and COL5A2 were significantly increased in ESCC samples (*p* < 0.001).

## Discussion

ESCC is a malignant tumor that poses a serious threat to human health due to its high incidence rate and low 5-year survival rate. Although numerous studies have investigated the mechanisms underlying ESCC, effective biomarkers for the diagnosis, prognosis and therapeutic targeting of ESCC remain scarce, and the mechanisms governing ESCC have not been fully elucidated [27, 28]. In the present study, six high-quality GEO datasets were selected to identify the hub genes associated with ESCC, as well as their associated biological pathways by integrated bioinformatic analysis. Finally, 720 DEGs consisting of 302 upregulatedgenes and 418 downregulated genes were identified, and they were significantly enriched in the cellular component of extracellular matrix component followed by the biological process of cell cycle phase and nuclear division. The primary enriched pathways were the cell cycle (hsa04110) and DNA replication (hsa03030). The top four genes were identified as hub genes based on the degree of connectivity in the PPI network, and these genes were validated in the TCGA database. The expression levels of these hub genes all showed notably elevated expression in ESCC samples compared with normal samples, and ESCC patients with upregulation of all four hub genes exhibited worse disease free survival.

GO analysis of the DEGs demonstrated that they were significantly enriched in the CC of the extracellular matrix (ECM) component (GO:0044420) and the BP of cell cycle phase (GO:0022403). Previous studies have shown that ECM remodeling not only promotes cancer development but is also associated with a poor prognosis in ESCC patients [29]. In keeping with ESCC’s metastatic propensity and high invasiveness, we found that the upregulated DEGs were significantly enriched in the ECM process. In addition, aberrant cell cycle progression has become one of the prominent features of various tumor cells [30]. It has been demonstrated that cycle-related genes in EC patients are significantly associated with lymph node metastasis and are not conducive to survival [31]. Deng et al. demonstrated that cinobufagin promoted cell cycle arrest and apoptosis via the p73 signaling pathway to prevent the growth of human ESCC cells [32]. Lu et al. observed that dracorhodin perchlorate could inhibit JAK2/STAT3 and AKT/FOXO3A pathways to induce apoptosis and G2/M cell cycle arrest in human ESCCs [33]. In this study, the expression levels of genes related to the cell cycle and mitotic regulation in patients with ESCC, such as CCNA1, CDK1, KIF23, and TPX2, were significantly altered, indicating that these genes might be crucial to ESCC development. Meanwhile, KEGG pathway analysis also confirmed these results. The upregulated genes were significantly enriched in the cell cycle (hsa04110) and DNA replication (hsa03030). These results were also consistent with the findings of a recent study, in which three modules from the PPI network were primarily related to such phenomena as DNA replication, the cell cycle and EMT [19]. These results may help to establish a foundation for further research investigating the biological processes and mechanisms involved in the development of ESCC.

In keeping with the results of the GO and KEGG analyses, four genes were considered to be hub genes in the PPI network, and their expression characteristics were also verified by the TCGA database. Osteopontin (SPP1) is a multifunctional 34 kDa extracellular matrix protein that plays important roles in adhesion and migration. Currently, SPP1 is considered to affect the occurrence and metastasis of various tumors [34]. Xu et al. demonstrated that inhibiting SPP1 expression inhibited proliferation and migration by activating ERK1/2 in ECA-109 cells [35], suggesting that SPP1 may play an important role in ESCC. Xing et al. indicated that the expression of SPP1 was notably elevated in ESCC patients compared with healthy controls through RNA transcriptome sequencing, indicating that SPP1 could serve as a serum biomarker for the detection of ESCC [12]. Meanwhile, SPP1 was identified as one of the predictive and prognostic factors for ESCC. Further analysis demonstrated that differentially expressed immune signatures in ESCC might be crucial to tumorigenesis and development by activating T cell and NF-kappa B signaling pathways [36]. Recently, SPP1 expression was reported to be associated with poor prognosis in locally advanced ESCC patients receiving preoperative chemoradiotherapy [37]. A meta-analysis involving 811 patients showed that overexpression of SPP1 might be a promising independent prognostic risk factor for ESCC patients in China and Japan [9].

To date, the expression of collagen family members has been observed to be abnormal in several cancers, such as breast and lung cancers [38–40]. COL10A1 and COL5A2 are members of the collagen family, and the dysregulation of COL10A1 and COL5A2 may represent a basis for cancer invasion and migration. The ectopic expression of COL10A1 and COL5A2 may affect the development of cancer, leading to genetic mutations and epigenetic alterations. Further analysis showed that these genes could activate ECM remodeling and the EMT, VEGFR3 and Wnt signaling pathways, which are oncogenic signaling pathways or processes. However, little research has investigated their crucial role in ESCC. Karagoz et al. employed proteomic and metabolic strategies anddemonstrated that 51 genes were differentially expressed between 91 ESCC tumor samples in five GEO datasets compared with normal tissue, indicating that these genes, including COL10A1, may act as specific biomarkers in ESCC [13]. Based on an integrated bioinformatic strategy, Yang et al. identified COL5A2 as a hub gene that was closely related to the survival of ESCC patients [19]. These results suggest that an in-depth study on the role played by collagen family members in ESCC is important for improved detection and treatment of ESCC in the future.

Increasing numbers of studies have obtained contradictory results regarding the function of human macrophage metalloelastase (also known as matrix metalloproteinase, MMP) in tumors. Ding et al. found that MMP12 is mainly located in tumor cells, suggesting that MMP12 was an impact factor in the progression of ESCC; however, MMP12 was not determined to be an independent prognostic factor [41]. Warnecke-Eberz et al. demonstrated that MMP12 was one of the diagnostic marker signatures for ESCC by transcriptome analysis [42]. Recent studies indicated that reductions in anion exchanger 2 (AE2) could activate MMP signaling pathways and enhance cellular movement in ESCC. Further analysis showed that AE2 was crucial to the poor prognosis of patients with ESCC [43]. Subsequently, Han et al. found that MMP12 was closely related to nodal metastasis, tumor grade and staged poor survival of ESCC owing to its high expression in tumor cells [44]. These studies demonstrated that MMP-mediated degradation of the ECM is essential to tumor invasion and metastasis in ESCC. However, the results regarding the function of MMP12 remain contradictory concerning ESCC progression. Therefore, to develop a novel therapeutic for ESCC, the function and mechanism of MMP12 require further analysis.

In the present study, using integrated bioinformatic analysis, we identified four hub genes involved in ESCC. These hub genes may be utilized not only in research on the molecular mechanisms governing ESCC but also as potential prognostic biomarkers for this cancer. However, the relationship between the hub genes and ESCC progression may be unreliable because this study was based on bioinformatic analysis of published data with a relatively small number of samples, and the hub genes were validated only with TCGA data and qPCR assays. Therefore, in-depth studies to obtain various forms of experimental validation should be undertaken with a large number of samples.

## Conclusions

Using integrated bioinformatic analysis, 720 DEGs were identified, consisting of 302 upregulated DEGs and 418 downregulated DEGs, and these genes were significantly enriched in the cellular component of the extracellular matrix followed by the biological process of the cell cycle phase and nuclear division. Four hub genes were identified that might play important roles in ESCC, namely, SPP1, MMP12, COL10A1 and COL5A2. The results of this study may help to elucidate the development and molecular mechanisms of ESCC, and it may also help us to identify candidate targets for the early detection and treatment of ESCC.

## Supplementary Information

**Additional file 1: Table S1.** Upregulated and down regulated genes in GEO datasets.

**Additional file 2: Table S2.** Go analysis for up and down-regulated DEGs, respectively.

**Additional file 3: Table S3.** KEGG analysis for up and down-regulated DEGs, respectively.

## Data Availability

The datasets used and/or analyzed during the current study are available from the corresponding author on reasonable request.

## Abbreviations

ESCC: Esophageal squamous cell carcinoma
EC: Esophageal cancer
DEGs: Differentially expressed genes
GEO: Gene expression omnibus
PPI: Protein–protein interaction
qRT-PCR: Real-time quantitative PCR
TCGA: The cancer genome atlas
KEGG: Kyotoencyclopedia of genes and genomes
GO: Gene ontology
STRING: Search tool for the retrieval of interacting genes
MCODE: Molecular complex detection
DAVID: Database for annotation, visualization and integrated discovery
SD: Standard deviation
MF: Molecular function
BP: Biological process
CC: Cellular component
ECM: Extracellular matrix
MMP: Matrix metalloproteinase
AE2: Anion exchanger 2
