# Supplementary material for: Identification of four genes and biological characteristics of esophageal squamous cell carcinoma by integrated bioinformatics analysis
Source: Cancer Cell Int. 2021 Feb 18;21:123. doi: 10.1186/s12935-021-01814-1 (PMC7890804; doi:10.1186/s12935-021-01814-1)
Supplement: Supplementary file 1 — Additional file 1: Table S1. Upregulated and down regulated genes in GEO datasets. [file 12935_2021_1814_MOESM1_ESM.docx]

**Additional file 1: Table S1 Upregulated and down regulated genes in GEO datasets**

| Name | P-value | adjPvalue | Log2FC |
| --- | --- | --- | --- |
| **MMP1** | 5.66E-26 | 1.23E-21 | 6.021246 |
| **MMP12** | 1.08E-17 | 2.35E-13 | 3.912592 |
| **MMP10** | 1.38E-17 | 3.01E-13 | 3.406641 |
| **SPP1** | 3.58E-16 | 7.78E-12 | 3.854737 |
| **PTHLH** | 5.60E-15 | 1.22E-10 | 3.180387 |
| **KRT17** | 1.18E-14 | 2.57E-10 | 3.28419 |
| **MAGEA6** | 3.66E-14 | 7.97E-10 | 2.776854 |
| **MMP3** | 4.16E-14 | 9.05E-10 | 2.942798 |
| **NELL2** | 1.96E-13 | 4.26E-09 | 2.894475 |
| **CXCL1** | 2.16E-13 | 4.70E-09 | 2.264962 |
| **MFAP2** | 2.48E-13 | 5.39E-09 | 2.675294 |
| **COL1A2** | 3.87E-13 | 8.43E-09 | 2.60471 |
| **LAMC2** | 4.64E-13 | 1.01E-08 | 2.041401 |
| **TGFBI** | 5.12E-13 | 1.11E-08 | 2.496093 |
| **MARCKSL1** | 1.27E-12 | 2.77E-08 | 2.184885 |
| **ANO1** | 1.99E-12 | 4.33E-08 | 2.620457 |
| **MFHAS1** | 2.50E-12 | 5.44E-08 | 2.163501 |
| **COL5A2** | 6.23E-12 | 1.35E-07 | 2.574901 |
| **APOBEC3B** | 7.15E-12 | 1.56E-07 | 2.348204 |
| **COL11A1** | 8.32E-12 | 1.81E-07 | 3.114278 |
| **CXCL8** | 8.55E-12 | 1.86E-07 | 2.389408 |
| **ISG15** | 8.55E-12 | 1.86E-07 | 2.38654 |
| **PPFIA1** | 1.42E-11 | 3.08E-07 | 1.878858 |
| **LPCAT1** | 1.79E-11 | 3.89E-07 | 2.04972 |
| **MMP13** | 2.27E-11 | 4.93E-07 | 2.900887 |
| **SNX10** | 2.39E-11 | 5.21E-07 | 1.994645 |
| **EPCAM** | 5.23E-11 | 1.14E-06 | 2.239009 |
| **COL1A1** | 7.05E-11 | 1.53E-06 | 1.958184 |
| **HLTF** | 7.31E-11 | 1.59E-06 | 1.590005 |
| **COL6A3** | 8.12E-11 | 1.77E-06 | 2.08601 |
| **DFNA5** | 8.59E-11 | 1.87E-06 | 2.077176 |
| **ARPC1B** | 8.74E-11 | 1.90E-06 | 1.604657 |
| **MIR1908** | 9.22E-11 | 2.01E-06 | 1.836406 |
| **INHBA** | 9.38E-11 | 2.04E-06 | 2.346431 |
| **COL10A1** | 9.39E-11 | 2.04E-06 | 2.399716 |
| **KIF14** | 1.13E-10 | 2.46E-06 | 1.995363 |
| **PMEPA1** | 1.17E-10 | 2.55E-06 | 1.928507 |
| **IGF2BP2** | 1.34E-10 | 2.91E-06 | 1.890294 |
| **LAMB3** | 1.36E-10 | 2.96E-06 | 1.887505 |
| **IGFBP3** | 1.42E-10 | 3.08E-06 | 1.985291 |
| **ODC1** | 1.52E-10 | 3.32E-06 | 2.311889 |
| **SOX4** | 1.57E-10 | 3.42E-06 | 1.554783 |
| **BID** | 1.73E-10 | 3.77E-06 | 1.912468 |
| **SERPINH1** | 1.99E-10 | 4.32E-06 | 1.959434 |
| **MEST** | 2.39E-10 | 5.20E-06 | 2.146799 |
| **PLAU** | 2.43E-10 | 5.28E-06 | 1.970456 |
| **CALB1** | 2.84E-10 | 6.17E-06 | 1.774166 |
| **LOC101928615** | 3.02E-10 | 6.57E-06 | 1.872481 |
| **VCAN** | 3.06E-10 | 6.65E-06 | 2.263402 |
| **HEY1** | 3.06E-10 | 6.65E-06 | 1.899422 |
| **SLCO1B3** | 3.38E-10 | 7.36E-06 | 1.980922 |
| **TRIP13** | 3.43E-10 | 7.46E-06 | 1.837908 |
| **TMEM97** | 3.43E-10 | 7.46E-06 | 1.516285 |
| **RAI14** | 4.55E-10 | 9.90E-06 | 1.852305 |
| **RPL39L** | 5.09E-10 | 1.11E-05 | 1.629783 |
| **GPNMB** | 5.58E-10 | 1.21E-05 | 1.944815 |
| **COL5A1** | 6.02E-10 | 1.31E-05 | 2.03956 |
| **PFN2** | 6.42E-10 | 1.40E-05 | 2.023018 |
| **COL7A1** | 7.19E-10 | 1.56E-05 | 1.47636 |
| **SNORA11E** | 7.28E-10 | 1.58E-05 | 1.849251 |
| **COL4A1** | 7.70E-10 | 1.67E-05 | 1.970918 |
| **SLC7A11** | 8.43E-10 | 1.83E-05 | 1.539327 |
| **IFI6** | 8.45E-10 | 1.84E-05 | 1.783476 |
| **SERPINE1** | 9.28E-10 | 2.02E-05 | 1.725714 |
| **MCM2** | 1.01E-09 | 2.20E-05 | 1.598149 |
| **CST1** | 1.03E-09 | 2.23E-05 | 2.412081 |
| **CDH3** | 1.14E-09 | 2.47E-05 | 1.916698 |
| **ITGA6** | 1.27E-09 | 2.77E-05 | 1.684371 |
| **CEP55** | 1.31E-09 | 2.85E-05 | 1.747964 |
| **CDKN3** | 1.34E-09 | 2.92E-05 | 1.774387 |
| **FADD** | 1.37E-09 | 2.99E-05 | 1.601796 |
| **LRRC8D** | 1.45E-09 | 3.16E-05 | 1.38212 |
| **FOXM1** | 1.55E-09 | 3.37E-05 | 1.767899 |
| **COL3A1** | 1.57E-09 | 3.41E-05 | 1.721847 |
| **CCL20** | 1.59E-09 | 3.46E-05 | 1.524457 |
| **KCNS3** | 1.82E-09 | 3.96E-05 | 1.661557 |
| **CDC6** | 1.96E-09 | 4.27E-05 | 1.672666 |
| **HOXB7** | 2.15E-09 | 4.69E-05 | 2.154343 |
| **MAGEA11** | 2.18E-09 | 4.73E-05 | 1.891314 |
| **MTERF3** | 2.61E-09 | 5.69E-05 | 1.273518 |
| **FST** | 2.79E-09 | 6.06E-05 | 1.7082 |
| **MMP9** | 3.21E-09 | 6.99E-05 | 1.879719 |
| **TOP2A** | 3.21E-09 | 6.99E-05 | 1.661465 |
| **GINS1** | 3.29E-09 | 7.15E-05 | 1.559212 |
| **MLF1** | 3.37E-09 | 7.34E-05 | 1.496552 |
| **LRP12** | 3.44E-09 | 7.48E-05 | 1.595463 |
| **RBP1** | 3.53E-09 | 7.67E-05 | 1.461298 |
| **DTL** | 3.69E-09 | 8.03E-05 | 1.758086 |
| **LIPG** | 3.82E-09 | 8.30E-05 | 1.213409 |
| **SLC25A32** | 4.08E-09 | 8.87E-05 | 1.452478 |
| **SULF1** | 4.11E-09 | 8.95E-05 | 1.833843 |
| **CDC25B** | 4.41E-09 | 9.60E-05 | 1.587554 |
| **BAMBI** | 4.68E-09 | 0.00010177 | 1.874121 |
| **LUM** | 5.36E-09 | 0.000116596 | 1.726852 |
| **UCHL1** | 6.07E-09 | 0.000132159 | 1.827599 |
| **PRAME** | 6.21E-09 | 0.000135205 | 1.201757 |
| **MINPP1** | 6.27E-09 | 0.000136381 | 1.419358 |
| **TMEM184B** | 6.95E-09 | 0.000151179 | 1.15314 |
| **TNFRSF12A** | 7.25E-09 | 0.000157727 | 1.3016 |
| **PXDN** | 7.73E-09 | 0.000168263 | 1.928245 |
| **KIF4A** | 8.06E-09 | 0.000175276 | 1.910829 |
| **PLOD3** | 8.43E-09 | 0.000183292 | 1.580286 |
| **SLC39A4** | 8.57E-09 | 0.000186334 | 1.291693 |
| **FBN2** | 8.85E-09 | 0.000192545 | 1.365172 |
| **PBK** | 9.03E-09 | 0.000196423 | 1.436967 |
| **ALCAM** | 9.03E-09 | 0.000196423 | 1.582025 |
| **LGALS1** | 9.38E-09 | 0.000204019 | 2.170065 |
| **ENAH** | 9.92E-09 | 0.000215853 | 1.545229 |
| **SNAPC1** | 1.23E-08 | 0.000266788 | 1.505652 |
| **MAGEA12** | 1.26E-08 | 0.000273636 | 1.273599 |
| **GALNT6** | 1.29E-08 | 0.000281576 | 1.945692 |
| **FAT1** | 1.33E-08 | 0.000290329 | 1.589658 |
| **UBE2C** | 1.37E-08 | 0.000298898 | 1.587114 |
| **ENO2** | 1.40E-08 | 0.000304167 | 1.67052 |
| **MIR8071-2** | 1.40E-08 | 0.000305538 | 2.375719 |
| **CTHRC1** | 1.55E-08 | 0.000337091 | 2.580395 |
| **VOPP1** | 1.62E-08 | 0.000352933 | 1.378518 |
| **BGN** | 1.63E-08 | 0.00035501 | 1.401012 |
| **TIMP1** | 1.75E-08 | 0.000379681 | 1.82531 |
| **FSCN1** | 1.77E-08 | 0.000386058 | 1.648385 |
| **HOXD11** | 1.80E-08 | 0.000391053 | 1.454327 |
| **SLC38A6** | 1.86E-08 | 0.000405473 | 1.440633 |
| **NEFL** | 1.90E-08 | 0.000414335 | 1.169949 |
| **SAC3D1** | 2.03E-08 | 0.000441897 | 1.1643 |
| **DHCR7** | 2.09E-08 | 0.00045466 | 1.278923 |
| **HMGB3** | 2.16E-08 | 0.00046948 | 1.383201 |
| **CDH11** | 2.45E-08 | 0.000533914 | 1.585742 |
| **HOMER3** | 2.52E-08 | 0.000548813 | 1.280967 |
| **TNFAIP6** | 2.90E-08 | 0.00063047 | 1.615847 |
| **THAP12** | 2.91E-08 | 0.00063284 | 1.085656 |
| **GMNN** | 2.93E-08 | 0.000637095 | 1.315465 |
| **LAMP3** | 3.19E-08 | 0.000694607 | 1.454113 |
| **GREM1** | 3.31E-08 | 0.000720721 | 1.744694 |
| **POSTN** | 3.36E-08 | 0.000731354 | 1.917167 |
| **TPX2** | 3.56E-08 | 0.000775147 | 1.582535 |
| **FZD6** | 3.67E-08 | 0.000797815 | 1.361287 |
| **RFC4** | 3.68E-08 | 0.00080045 | 1.648353 |
| **NETO2** | 3.81E-08 | 0.000827963 | 1.570349 |
| **JAG2** | 4.06E-08 | 0.000882475 | 1.138496 |
| **MRGBP** | 4.11E-08 | 0.000893729 | 1.226756 |
| **MDK** | 4.13E-08 | 0.0008994 | 1.210531 |
| **AMIGO2** | 4.47E-08 | 0.00097175 | 1.295938 |
| **TEAD4** | 4.54E-08 | 0.000988139 | 1.199744 |
| **MYH10** | 4.63E-08 | 0.001006734 | 1.037114 |
| **BLM** | 4.77E-08 | 0.001038373 | 1.53831 |
| **FJX1** | 5.17E-08 | 0.001124536 | 1.333784 |
| **MIR7110** | 5.17E-08 | 0.001124536 | 1.377961 |
| **DNMT3B** | 5.59E-08 | 0.001216575 | 1.82432 |
| **PLAUR** | 5.59E-08 | 0.001216575 | 1.322118 |
| **SLC2A1** | 5.63E-08 | 0.001223908 | 1.118895 |
| **AKR1B1** | 6.26E-08 | 0.001362328 | 1.508645 |
| **LAPTM4B** | 6.30E-08 | 0.001370385 | 1.528628 |
| **FAM64A** | 6.37E-08 | 0.001386618 | 1.388714 |
| **SSFA2** | 6.56E-08 | 0.001427904 | 1.391996 |
| **LAMB1** | 6.65E-08 | 0.001446759 | 1.314622 |
| **HOXA10** | 6.68E-08 | 0.001453164 | 1.904021 |
| **PFDN2** | 6.96E-08 | 0.001513554 | 1.033007 |
| **HOXD10** | 7.37E-08 | 0.001603443 | 1.662093 |
| **TRAM2** | 7.41E-08 | 0.001612672 | 1.167014 |
| **FN1** | 7.45E-08 | 0.001620542 | 1.459678 |
| **HOXC10** | 7.54E-08 | 0.001640836 | 1.758796 |
| **ZNF281** | 7.89E-08 | 0.00171714 | 1.135126 |
| **RAD51AP1** | 8.17E-08 | 0.00177646 | 1.632104 |
| **HSPBAP1** | 8.54E-08 | 0.001858201 | 1.234251 |
| **NUAK1** | 9.06E-08 | 0.00197041 | 1.602439 |
| **COL4A2** | 9.17E-08 | 0.001994129 | 1.743097 |
| **DNMT1** | 9.44E-08 | 0.002053634 | 1.149163 |
| **CTTN** | 9.49E-08 | 0.002064975 | 1.03218 |
| **THY1** | 9.50E-08 | 0.002066659 | 1.304568 |
| **IFI35** | 9.70E-08 | 0.002110862 | 1.376411 |
| **NUSAP1** | 9.86E-08 | 0.002145831 | 1.2078 |
| **BRIX1** | 1.02E-07 | 0.002229309 | 1.000203 |
| **ASPM** | 1.04E-07 | 0.002265904 | 1.471721 |
| **ECT2** | 1.08E-07 | 0.002343028 | 1.7834 |
| **TNC** | 1.11E-07 | 0.002405702 | 1.36806 |
| **SPARC** | 1.12E-07 | 0.002433516 | 1.383534 |
| **RUVBL1** | 1.18E-07 | 0.002576461 | 1.18109 |
| **TMEM158** | 1.18E-07 | 0.002576461 | 1.474318 |
| **KRT8** | 1.22E-07 | 0.002660226 | 1.195685 |
| **CKS1B** | 1.23E-07 | 0.002665395 | 1.515423 |
| **KIF18B** | 1.24E-07 | 0.002695578 | 1.186788 |
| **XCL2** | 1.26E-07 | 0.002731193 | 1.720264 |
| **PRSS23** | 1.30E-07 | 0.002819052 | 1.299275 |
| **BST2** | 1.31E-07 | 0.002848019 | 1.046661 |
| **PGBD5** | 1.34E-07 | 0.002907975 | 1.814381 |
| **NCAPG** | 1.35E-07 | 0.002938345 | 1.126983 |
| **NCAPH** | 1.36E-07 | 0.002968979 | 1.287894 |
| **DDX60** | 1.37E-07 | 0.002979704 | 1.425005 |
| **CYP24A1** | 1.41E-07 | 0.003062483 | 1.32237 |
| **ITGA3** | 1.44E-07 | 0.003126172 | 1.224666 |
| **BUB1B** | 1.46E-07 | 0.003182048 | 1.311494 |
| **IQCJ-SCHIP1** | 1.46E-07 | 0.003182048 | 1.333018 |
| **NCAPD2** | 1.54E-07 | 0.00335892 | 1.082137 |
| **DLGAP5** | 1.56E-07 | 0.003395218 | 1.536462 |
| **AIM2** | 1.60E-07 | 0.00347894 | 1.096737 |
| **IL36G** | 1.61E-07 | 0.003505995 | 1.250955 |
| **NFE2L3** | 1.74E-07 | 0.003788259 | 1.299127 |
| **CXCL6** | 1.78E-07 | 0.003864275 | 1.524531 |
| **HTRA1** | 1.89E-07 | 0.004104005 | 1.408832 |
| **PUS7** | 1.90E-07 | 0.004140419 | 1.119271 |
| **BCAT1** | 1.96E-07 | 0.004263698 | 1.350189 |
| **ACTL6A** | 2.09E-07 | 0.004541325 | 1.210757 |
| **UBE2S** | 2.20E-07 | 0.004780798 | 1.169559 |
| **ATAD2** | 2.22E-07 | 0.0048289 | 1.524608 |
| **DLX5** | 2.34E-07 | 0.005093491 | 1.134631 |
| **TLR2** | 2.37E-07 | 0.005166282 | 1.102468 |
| **HMMR** | 2.38E-07 | 0.005176541 | 1.104067 |
| **CTSC** | 2.41E-07 | 0.005239939 | 1.24083 |
| **EN1** | 2.47E-07 | 0.005364644 | 1.489363 |
| **KIF20A** | 2.54E-07 | 0.005521548 | 1.401148 |
| **MRPS17** | 2.61E-07 | 0.005674062 | 1.083421 |
| **PROCR** | 2.62E-07 | 0.005700504 | 1.186995 |
| **CDC20** | 2.67E-07 | 0.005818779 | 1.384384 |
| **MYO1B** | 2.86E-07 | 0.00622181 | 1.02637 |
| **SLC39A6** | 2.89E-07 | 0.00628116 | 1.04612 |
| **LBH** | 2.89E-07 | 0.00628116 | 1.282924 |
| **DSG2** | 2.94E-07 | 0.006400522 | 1.342724 |
| **PRSS21** | 3.03E-07 | 0.006584727 | 1.433317 |
| **LOXL1** | 3.14E-07 | 0.006835917 | 1.315145 |
| **TTK** | 3.25E-07 | 0.00706468 | 1.402659 |
| **PTPRK** | 3.37E-07 | 0.00732194 | 1.135775 |
| **SMYD3** | 3.54E-07 | 0.007705597 | 1.336776 |
| **MAGEA2B** | 3.55E-07 | 0.007722438 | 1.14652 |
| **KIF18A** | 3.68E-07 | 0.007999448 | 1.186456 |
| **CCDC86** | 3.73E-07 | 0.008105438 | 1.061122 |
| **AHR** | 3.74E-07 | 0.008135671 | 1.295459 |
| **WDHD1** | 3.81E-07 | 0.008284689 | 1.113528 |
| **LRP8** | 3.91E-07 | 0.008508514 | 1.076829 |
| **EFNA1** | 4.05E-07 | 0.008816502 | 1.216375 |
| **SNAI2** | 4.07E-07 | 0.008856087 | 1.546947 |
| **MAGEA4** | 4.08E-07 | 0.008880647 | 1.23661 |
| **THBS2** | 4.16E-07 | 0.009054983 | 1.618932 |
| **SOAT1** | 4.17E-07 | 0.009074048 | 1.063593 |
| **IFI44L** | 4.35E-07 | 0.009462783 | 1.53023 |
| **MYBL2** | 4.39E-07 | 0.009552574 | 1.411914 |
| **SLC16A1** | 4.52E-07 | 0.009841295 | 1.20433 |
| **SPAG5** | 5.03E-07 | 0.010949139 | 1.369868 |
| **APOC1** | 5.08E-07 | 0.011042288 | 1.37115 |
| **LY96** | 5.09E-07 | 0.011069994 | 1.340166 |
| **SH3BP4** | 5.12E-07 | 0.011136069 | 1.073867 |
| **ACVR1** | 5.29E-07 | 0.011517569 | 1.158519 |
| **COL16A1** | 5.33E-07 | 0.011586426 | 1.137076 |
| **SPC25** | 5.48E-07 | 0.0119254 | 1.398541 |
| **KNTC1** | 5.76E-07 | 0.012525719 | 1.121919 |
| **PARP12** | 6.24E-07 | 0.013584671 | 1.158066 |
| **NCF2** | 6.40E-07 | 0.013918717 | 1.173358 |
| **FAP** | 6.45E-07 | 0.014031511 | 1.372188 |
| **SLC20A1** | 6.99E-07 | 0.015205793 | 1.097066 |
| **PTDSS1** | 7.56E-07 | 0.016445038 | 1.080947 |
| **IRS1** | 7.68E-07 | 0.016703516 | 1.000972 |
| **POPDC3** | 7.69E-07 | 0.016738245 | 1.365002 |
| **MAGEA9B** | 7.73E-07 | 0.016811743 | 1.341186 |
| **TMEM45A** | 7.80E-07 | 0.016965226 | 1.206764 |
| **PPT1** | 7.98E-07 | 0.017363916 | 1.057552 |
| **DBF4** | 8.09E-07 | 0.017610048 | 1.115527 |
| **NUDT11** | 8.11E-07 | 0.017633836 | 1.493714 |
| **FARP1** | 8.38E-07 | 0.018229292 | 1.035116 |
| **BCL2A1** | 8.67E-07 | 0.018866576 | 1.029671 |
| **CENPE** | 8.81E-07 | 0.019155714 | 1.255698 |
| **NTS** | 8.93E-07 | 0.019426799 | 1.864914 |
| **CCNA1** | 9.09E-07 | 0.019784248 | 1.249859 |
| **MCM7** | 9.15E-07 | 0.019915956 | 1.127071 |
| **PITX2** | 9.26E-07 | 0.020146594 | 1.516471 |
| **TFRC** | 9.26E-07 | 0.020146594 | 1.381124 |
| **ORC6** | 9.28E-07 | 0.020196946 | 1.207064 |
| **PRC1** | 9.43E-07 | 0.020521735 | 1.271831 |
| **COCH** | 9.46E-07 | 0.020580058 | 1.238421 |
| **TUSC3** | 9.57E-07 | 0.020812818 | 1.024717 |
| **HJURP** | 9.65E-07 | 0.020985644 | 1.051718 |
| **ATP2B1** | 9.86E-07 | 0.02144426 | 1.155512 |
| **BORA** | 1.06E-06 | 0.023008183 | 1.086033 |
| **FAM60A** | 1.10E-06 | 0.023947929 | 1.061061 |
| **P3H1** | 1.18E-06 | 0.025732221 | 1.041484 |
| **ITGAV** | 1.18E-06 | 0.025763469 | 1.123493 |
| **CDK1** | 1.20E-06 | 0.026101477 | 1.387808 |
| **MLLT11** | 1.32E-06 | 0.028788395 | 1.112773 |
| **HOXA10-HOXA9** | 1.33E-06 | 0.028966954 | 1.678008 |
| **CTSK** | 1.39E-06 | 0.030240055 | 1.097127 |
| **POLE2** | 1.40E-06 | 0.03044944 | 1.004326 |
| **NDC80** | 1.42E-06 | 0.030871679 | 1.232486 |
| **CXCL10** | 1.42E-06 | 0.030929303 | 1.357878 |
| **TWIST1** | 1.44E-06 | 0.031298576 | 1.50885 |
| **KRT14** | 1.46E-06 | 0.031769551 | 1.131922 |
| **NID2** | 1.47E-06 | 0.031880601 | 1.33526 |
| **FANCI** | 1.49E-06 | 0.03232804 | 1.251748 |
| **MTCL1** | 1.51E-06 | 0.032894688 | 1.174992 |
| **P3H4** | 1.55E-06 | 0.03381853 | 1.580861 |
| **SFRP4** | 1.64E-06 | 0.035706776 | 1.219258 |
| **PLA2G7** | 1.68E-06 | 0.03655517 | 1.233359 |
| **RNASEH2A** | 1.71E-06 | 0.037289719 | 1.004775 |
| **RAB15** | 1.81E-06 | 0.039295184 | 1.260912 |
| **ERVMER34-1** | 1.83E-06 | 0.039827692 | 1.335937 |
| **ADORA2B** | 1.84E-06 | 0.040084441 | 1.075733 |
| **NEK2** | 1.87E-06 | 0.040610047 | 1.093932 |
| **PLTP** | 1.93E-06 | 0.04194814 | 1.096076 |
| **LOX** | 1.94E-06 | 0.042219928 | 1.046381 |
| **COL4A5** | 1.95E-06 | 0.04249312 | 1.029386 |
| **HACD3** | 1.96E-06 | 0.042581313 | 1.087928 |
| **PLXNA1** | 2.18E-06 | 0.047471052 | 1.017446 |
| **KIF23** | 2.20E-06 | 0.047957481 | 1.261979 |
| **IFITM1** | 2.23E-06 | 0.048563782 | 1.135689 |
| **NMB** | 2.25E-06 | 0.048882925 | 1.057691 |
| **Name** | **Pvalue** | **Adj Pvalue** | **logFC** |
| **CRISP3** | 8.84E-22 | 1.92E-17 | -6.62051 |
| **CRNN** | 2.64E-21 | 5.74E-17 | -6.19497 |
| **MAL** | 1.00E-19 | 2.18E-15 | -5.68186 |
| **SPINK5** | 9.50E-19 | 2.07E-14 | -4.53561 |
| **ENDOU** | 1.93E-18 | 4.19E-14 | -4.45193 |
| **CLCA4** | 1.93E-18 | 4.19E-14 | -4.92134 |
| **TGM3** | 7.31E-17 | 1.59E-12 | -5.13577 |
| **TMPRSS11E** | 1.99E-16 | 4.33E-12 | -4.56618 |
| **SLURP1** | 3.58E-16 | 7.78E-12 | -4.45583 |
| **CRCT1** | 3.58E-16 | 7.78E-12 | -4.94555 |
| **SCEL** | 1.75E-15 | 3.80E-11 | -4.34729 |
| **ECM1** | 4.68E-15 | 1.02E-10 | -3.50269 |
| **CYP4B1** | 1.01E-14 | 2.19E-10 | -3.60988 |
| **GABRP** | 1.72E-14 | 3.74E-10 | -3.36844 |
| **KLK13** | 2.63E-14 | 5.72E-10 | -3.01564 |
| **C2orf54** | 2.81E-14 | 6.12E-10 | -2.99719 |
| **FLG** | 3.43E-14 | 7.47E-10 | -3.85384 |
| **SCNN1B** | 3.90E-14 | 8.49E-10 | -3.00243 |
| **CLIC3** | 4.16E-14 | 9.05E-10 | -4.07401 |
| **PSCA** | 5.66E-14 | 1.23E-09 | -3.39292 |
| **HPGD** | 6.37E-14 | 1.39E-09 | -3.552 |
| **TGM1** | 6.76E-14 | 1.47E-09 | -3.29744 |
| **RHCG** | 7.16E-14 | 1.56E-09 | -3.53548 |
| **GDPD3** | 7.58E-14 | 1.65E-09 | -2.94752 |
| **PRSS3** | 8.03E-14 | 1.75E-09 | -2.87674 |
| **PPP1R3C** | 9.49E-14 | 2.06E-09 | -3.37952 |
| **SERPINB2** | 1.27E-13 | 2.76E-09 | -3.41897 |
| **GYS2** | 1.69E-13 | 3.68E-09 | -3.14047 |
| **CWH43** | 2.37E-13 | 5.17E-09 | -3.23011 |
| **EPB41L3** | 2.61E-13 | 5.67E-09 | -3.32691 |
| **KLK12** | 2.86E-13 | 6.22E-09 | -2.92901 |
| **FMO2** | 3.35E-13 | 7.29E-09 | -3.41122 |
| **CXCR2** | 3.74E-13 | 8.14E-09 | -3.67573 |
| **CYP2C18** | 4.45E-13 | 9.68E-09 | -2.64164 |
| **CEACAM7** | 4.84E-13 | 1.05E-08 | -3.17151 |
| **KRT13** | 5.12E-13 | 1.11E-08 | -3.42259 |
| **IL1RN** | 8.16E-13 | 1.77E-08 | -2.54215 |
| **KRT4** | 8.16E-13 | 1.77E-08 | -2.89306 |
| **PTK6** | 9.50E-13 | 2.07E-08 | -2.74406 |
| **UPK1A** | 9.86E-13 | 2.14E-08 | -3.13486 |
| **ANXA9** | 1.14E-12 | 2.48E-08 | -2.53276 |
| **EMP1** | 1.18E-12 | 2.58E-08 | -2.77399 |
| **CEACAM6** | 1.63E-12 | 3.54E-08 | -3.43624 |
| **FUT3** | 1.68E-12 | 3.66E-08 | -2.33612 |
| **MGLL** | 1.80E-12 | 3.92E-08 | -2.72391 |
| **GPX3** | 2.75E-12 | 5.98E-08 | -3.22182 |
| **BBOX1** | 3.71E-12 | 8.07E-08 | -3.22732 |
| **ZNF185** | 3.96E-12 | 8.62E-08 | -2.47012 |
| **RRAGD** | 4.45E-12 | 9.69E-08 | -2.35176 |
| **HLF** | 4.72E-12 | 1.03E-07 | -2.34362 |
| **CEACAM1** | 4.85E-12 | 1.06E-07 | -2.33939 |
| **IL18** | 4.99E-12 | 1.09E-07 | -3.00762 |
| **CRABP2** | 5.29E-12 | 1.15E-07 | -2.59037 |
| **CDA** | 6.96E-12 | 1.51E-07 | -2.30556 |
| **KLK11** | 7.34E-12 | 1.60E-07 | -2.4239 |
| **ACPP** | 8.83E-12 | 1.92E-07 | -2.47461 |
| **PPL** | 1.11E-11 | 2.41E-07 | -2.72529 |
| **SPRR2C** | 1.35E-11 | 2.93E-07 | -3.06129 |
| **MXD1** | 1.38E-11 | 3.01E-07 | -2.54349 |
| **CEACAM5** | 1.42E-11 | 3.08E-07 | -2.20874 |
| **TCN1** | 1.42E-11 | 3.08E-07 | -2.463 |
| **KAT2B** | 1.42E-11 | 3.08E-07 | -2.81061 |
| **EPS8L1** | 1.63E-11 | 3.55E-07 | -2.36302 |
| **GALNT12** | 1.79E-11 | 3.89E-07 | -2.34514 |
| **CYP3A5** | 1.83E-11 | 3.98E-07 | -2.31337 |
| **IL36A** | 1.87E-11 | 4.07E-07 | -2.84006 |
| **ABLIM3** | 1.87E-11 | 4.07E-07 | -2.27431 |
| **PLAC8** | 2.57E-11 | 5.59E-07 | -2.85626 |
| **PAX9** | 2.85E-11 | 6.19E-07 | -2.26396 |
| **TMPRSS11D** | 3.73E-11 | 8.11E-07 | -2.88419 |
| **ADH7** | 3.81E-11 | 8.28E-07 | -2.2561 |
| **TTC9** | 3.88E-11 | 8.45E-07 | -2.53612 |
| **SERPINB13** | 4.04E-11 | 8.80E-07 | -2.06925 |
| **SULT2B1** | 4.13E-11 | 8.98E-07 | -2.20455 |
| **C1orf116** | 4.83E-11 | 1.05E-06 | -2.38628 |
| **KLK7** | 5.22E-11 | 1.14E-06 | -2.03974 |
| **SERPINB1** | 5.42E-11 | 1.18E-06 | -2.3837 |
| **CYP2J2** | 5.53E-11 | 1.20E-06 | -2.24581 |
| **TP53I3** | 6.19E-11 | 1.35E-06 | -2.26333 |
| **SERPINB3** | 6.67E-11 | 1.45E-06 | -2.87237 |
| **EHD3** | 6.79E-11 | 1.48E-06 | -2.20175 |
| **CES2** | 7.72E-11 | 1.68E-06 | -1.89268 |
| **SYNPO2L** | 8.14E-11 | 1.77E-06 | -2.32707 |
| **KRT24** | 1.08E-10 | 2.35E-06 | -2.5176 |
| **CITED2** | 1.13E-10 | 2.46E-06 | -2.19234 |
| **EPHX2** | 1.25E-10 | 2.73E-06 | -2.03335 |
| **DHRS1** | 1.27E-10 | 2.77E-06 | -2.08294 |
| **BLNK** | 1.54E-10 | 3.36E-06 | -2.78037 |
| **OR7E14P** | 1.57E-10 | 3.42E-06 | -2.22316 |
| **SLC16A6** | 1.62E-10 | 3.54E-06 | -2.65441 |
| **SPRR3** | 1.73E-10 | 3.77E-06 | -3.08536 |
| **GPD1L** | 1.91E-10 | 4.15E-06 | -2.42653 |
| **SASH1** | 1.91E-10 | 4.15E-06 | -2.4033 |
| **GCNT3** | 2.46E-10 | 5.35E-06 | -1.87875 |
| **FUT6** | 2.65E-10 | 5.76E-06 | -1.94666 |
| **CH25H** | 2.93E-10 | 6.38E-06 | -1.96798 |
| **BEX4** | 3.06E-10 | 6.65E-06 | -2.50656 |
| **PTN** | 3.06E-10 | 6.66E-06 | -2.09792 |
| **TMPRSS11B** | 3.15E-10 | 6.84E-06 | -3.72963 |
| **HOPX** | 3.63E-10 | 7.89E-06 | -2.32436 |
| **DUSP5** | 3.68E-10 | 8.00E-06 | -2.63713 |
| **EPHX3** | 3.68E-10 | 8.00E-06 | -2.31204 |
| **NUCB2** | 3.73E-10 | 8.11E-06 | -2.17374 |
| **HSPB8** | 3.89E-10 | 8.46E-06 | -2.25818 |
| **S100P** | 4.09E-10 | 8.91E-06 | -2.25782 |
| **UBL3** | 4.34E-10 | 9.43E-06 | -2.27416 |
| **EVPL** | 4.76E-10 | 1.04E-05 | -2.04539 |
| **ALS2CL** | 4.89E-10 | 1.06E-05 | -1.54004 |
| **ZNF365** | 5.72E-10 | 1.24E-05 | -2.22622 |
| **SMAGP** | 6.84E-10 | 1.49E-05 | -1.93182 |
| **PITX1** | 7.00E-10 | 1.52E-05 | -2.28804 |
| **MALL** | 7.19E-10 | 1.56E-05 | -2.5494 |
| **SLC35C1** | 8.33E-10 | 1.81E-05 | -1.44798 |
| **MEIS1** | 8.74E-10 | 1.90E-05 | -1.5713 |
| **TMPRSS2** | 9.28E-10 | 2.02E-05 | -1.72121 |
| **ABLIM1** | 9.39E-10 | 2.04E-05 | -1.99013 |
| **SERPINB4** | 9.82E-10 | 2.14E-05 | -2.79133 |
| **TMEM40** | 1.04E-09 | 2.27E-05 | -1.75258 |
| **DIO2** | 1.06E-09 | 2.30E-05 | -1.91087 |
| **CNN3** | 1.13E-09 | 2.46E-05 | -1.79965 |
| **RNF39** | 1.15E-09 | 2.49E-05 | -1.65304 |
| **VAV3** | 1.16E-09 | 2.52E-05 | -1.81797 |
| **ANXA1** | 1.27E-09 | 2.76E-05 | -1.74359 |
| **CAPN5** | 1.45E-09 | 3.16E-05 | -1.51648 |
| **IVL** | 1.46E-09 | 3.19E-05 | -2.23486 |
| **PMM1** | 1.58E-09 | 3.45E-05 | -1.54525 |
| **ANXA3** | 1.62E-09 | 3.52E-05 | -1.97403 |
| **AQP3** | 1.63E-09 | 3.55E-05 | -2.00475 |
| **LPIN1** | 1.65E-09 | 3.60E-05 | -1.83475 |
| **PRSS3P2** | 1.76E-09 | 3.84E-05 | -1.40541 |
| **UPK3B** | 1.90E-09 | 4.13E-05 | -1.7615 |
| **SORT1** | 1.98E-09 | 4.31E-05 | -1.53363 |
| **TJP3** | 2.00E-09 | 4.36E-05 | -1.53331 |
| **USP6NL** | 2.18E-09 | 4.74E-05 | -1.64235 |
| **TMOD3** | 2.41E-09 | 5.25E-05 | -1.79986 |
| **YOD1** | 2.65E-09 | 5.77E-05 | -1.93724 |
| **FCER1A** | 2.72E-09 | 5.92E-05 | -2.22034 |
| **DNASE1L3** | 2.72E-09 | 5.92E-05 | -1.78028 |
| **PIM1** | 2.89E-09 | 6.28E-05 | -1.60465 |
| **EHF** | 2.92E-09 | 6.36E-05 | -2.1182 |
| **ALOX12** | 3.06E-09 | 6.66E-05 | -2.389 |
| **ETFDH** | 3.18E-09 | 6.93E-05 | -1.84664 |
| **CRYAB** | 3.28E-09 | 7.13E-05 | -1.70139 |
| **ALOX15B** | 3.44E-09 | 7.48E-05 | -1.545 |
| **CCNG2** | 3.44E-09 | 7.48E-05 | -2.01918 |
| **MIR6778** | 3.54E-09 | 7.70E-05 | -1.46475 |
| **SLC24A3** | 3.69E-09 | 8.03E-05 | -2.10217 |
| **NMU** | 4.04E-09 | 8.78E-05 | -2.02416 |
| **DSG1** | 4.22E-09 | 9.19E-05 | -2.37308 |
| **GNE** | 4.39E-09 | 9.55E-05 | -1.40034 |
| **TRIP10** | 5.07E-09 | 0.00011 | -1.78312 |
| **BSPRY** | 5.65E-09 | 0.000123 | -1.46837 |
| **CRIP2** | 5.75E-09 | 0.000125 | -1.66996 |
| **SLC39A2** | 6.21E-09 | 0.000135 | -1.86758 |
| **HLA-DQB2** | 6.49E-09 | 0.000141 | -1.57753 |
| **CYP4F12** | 7.01E-09 | 0.000152 | -1.59167 |
| **VLDLR** | 7.56E-09 | 0.000165 | -1.71158 |
| **PRSS2** | 8.15E-09 | 0.000177 | -1.24407 |
| **AIM1L** | 8.22E-09 | 0.000179 | -1.29116 |
| **SPINK7** | 8.49E-09 | 0.000185 | -3.22123 |
| **TJP1** | 8.57E-09 | 0.000186 | -1.60917 |
| **FAM189A2** | 8.85E-09 | 0.000193 | -1.54736 |
| **MGST2** | 9.37E-09 | 0.000204 | -1.40289 |
| **MIR4680** | 9.56E-09 | 0.000208 | -1.68429 |
| **CLTB** | 1.02E-08 | 0.000221 | -1.22504 |
| **EXPH5** | 1.07E-08 | 0.000234 | -1.31524 |
| **ERO1A** | 1.11E-08 | 0.000241 | -1.95579 |
| **MAFF** | 1.22E-08 | 0.000265 | -1.30727 |
| **NAGK** | 1.25E-08 | 0.000271 | -1.70338 |
| **ITM2A** | 1.45E-08 | 0.000315 | -1.69633 |
| **SAMD9** | 1.45E-08 | 0.000315 | -1.64281 |
| **AIM1** | 1.46E-08 | 0.000318 | -1.75413 |
| **IL22RA1** | 1.52E-08 | 0.00033 | -1.03217 |
| **ZNF426** | 1.58E-08 | 0.000343 | -1.67458 |
| **CYP4F3** | 1.59E-08 | 0.000345 | -1.7088 |
| **RAB11A** | 1.59E-08 | 0.000345 | -1.40383 |
| **RANBP9** | 1.69E-08 | 0.000367 | -1.78459 |
| **SLC26A2** | 1.75E-08 | 0.00038 | -1.39373 |
| **LYPD3** | 1.82E-08 | 0.000397 | -1.71997 |
| **IL13RA1** | 1.82E-08 | 0.000397 | -1.43766 |
| **TOB1** | 1.89E-08 | 0.000411 | -1.15866 |
| **MUC1** | 1.90E-08 | 0.000414 | -1.57283 |
| **PRR15L** | 1.96E-08 | 0.000426 | -1.19897 |
| **ADGRF1** | 1.99E-08 | 0.000433 | -1.57822 |
| **LCN2** | 2.06E-08 | 0.000447 | -2.48182 |
| **PHACTR2** | 2.08E-08 | 0.000451 | -1.37313 |
| **SLC13A4** | 2.09E-08 | 0.000455 | -1.32692 |
| **SPRR2B** | 2.13E-08 | 0.000464 | -2.4216 |
| **ID4** | 2.15E-08 | 0.000468 | -1.66297 |
| **SH3BGRL** | 2.18E-08 | 0.000474 | -1.28437 |
| **TF** | 2.23E-08 | 0.000484 | -1.53664 |
| **MPZL2** | 2.41E-08 | 0.000524 | -1.67108 |
| **DUOX1** | 2.42E-08 | 0.000527 | -1.36145 |
| **CSTB** | 2.45E-08 | 0.000534 | -1.7952 |
| **PADI1** | 2.58E-08 | 0.00056 | -2.17523 |
| **CDKN2AIP** | 2.61E-08 | 0.000568 | -1.38036 |
| **BARX2** | 2.76E-08 | 0.0006 | -1.1524 |
| **CD24** | 2.94E-08 | 0.00064 | -1.73157 |
| **RRAD** | 3.08E-08 | 0.000669 | -1.92619 |
| **CYP11A1** | 3.09E-08 | 0.000672 | -1.07638 |
| **PDLIM2** | 3.13E-08 | 0.000681 | -1.54349 |
| **EYA2** | 3.17E-08 | 0.00069 | -1.72396 |
| **NLRX1** | 3.26E-08 | 0.000708 | -1.33791 |
| **HCAR3** | 3.28E-08 | 0.000713 | -1.17837 |
| **PRDM1** | 3.37E-08 | 0.000732 | -1.20817 |
| **SELENBP1** | 3.45E-08 | 0.000751 | -1.15064 |
| **MANSC1** | 3.50E-08 | 0.000761 | -1.46748 |
| **NEBL** | 3.50E-08 | 0.000761 | -1.12453 |
| **MOSPD1** | 3.78E-08 | 0.000823 | -1.24581 |
| **PTGS1** | 3.81E-08 | 0.000828 | -1.27785 |
| **RAB25** | 3.88E-08 | 0.000845 | -1.56642 |
| **CAPN14** | 3.93E-08 | 0.000854 | -3.01186 |
| **ECHDC2** | 3.95E-08 | 0.00086 | -1.42172 |
| **ELOVL6** | 4.01E-08 | 0.000871 | -1.46299 |
| **XYLT1** | 4.11E-08 | 0.000894 | -1.14811 |
| **RIPK4** | 4.24E-08 | 0.000922 | -1.1755 |
| **NDRG2** | 4.46E-08 | 0.00097 | -1.2356 |
| **PLBD1** | 4.47E-08 | 0.000972 | -1.49456 |
| **PHACTR4** | 4.66E-08 | 0.001013 | -1.52983 |
| **PLLP** | 4.95E-08 | 0.001077 | -1.13037 |
| **CHAC1** | 5.11E-08 | 0.001111 | -1.32937 |
| **ESPL1** | 5.17E-08 | 0.001125 | -1.5431 |
| **GLTP** | 5.20E-08 | 0.001131 | -1.48707 |
| **NPEPPS** | 5.23E-08 | 0.001138 | -1.31663 |
| **PAIP2B** | 5.39E-08 | 0.001173 | -1.68583 |
| **UGT1A3** | 5.49E-08 | 0.001195 | -1.34505 |
| **SECISBP2L** | 5.53E-08 | 0.001202 | -1.25138 |
| **GCHFR** | 5.56E-08 | 0.001209 | -1.47118 |
| **ALDH3A2** | 5.63E-08 | 0.001224 | -1.08007 |
| **P2RY2** | 5.66E-08 | 0.001231 | -1.24911 |
| **EPS8L2** | 5.69E-08 | 0.001239 | -1.61933 |
| **C18orf25** | 5.69E-08 | 0.001239 | -1.42559 |
| **SLC27A6** | 5.73E-08 | 0.001246 | -1.79975 |
| **LDOC1** | 6.08E-08 | 0.001323 | -1.00681 |
| **BLVRB** | 6.08E-08 | 0.001323 | -1.28433 |
| **RORA** | 6.41E-08 | 0.001395 | -1.59868 |
| **CST6** | 6.64E-08 | 0.001445 | -1.44806 |
| **THSD4** | 6.72E-08 | 0.001462 | -1.16922 |
| **MYO6** | 6.76E-08 | 0.00147 | -1.3908 |
| **MYO5C** | 6.84E-08 | 0.001487 | -1.11254 |
| **ASCC2** | 7.17E-08 | 0.001561 | -1.26289 |
| **GAB2** | 7.20E-08 | 0.001567 | -1.14576 |
| **CBR3** | 7.41E-08 | 0.001613 | -1.08319 |
| **CAST** | 7.46E-08 | 0.001622 | -1.22762 |
| **EPB41L4A** | 7.50E-08 | 0.001631 | -1.12737 |
| **DYNLT3** | 7.63E-08 | 0.001659 | -1.42619 |
| **SIM2** | 7.72E-08 | 0.001679 | -1.72903 |
| **ATP6V0A4** | 7.94E-08 | 0.001727 | -1.37207 |
| **DHRS9** | 8.12E-08 | 0.001766 | -2.08949 |
| **MOXD1** | 8.49E-08 | 0.001848 | -1.08643 |
| **RASAL1** | 8.88E-08 | 0.001932 | -1.1666 |
| **AMOTL2** | 8.93E-08 | 0.001943 | -1.19173 |
| **SREK1IP1** | 9.39E-08 | 0.002042 | -1.00383 |
| **INPP1** | 9.61E-08 | 0.002091 | -1.49068 |
| **CUL4B** | 9.76E-08 | 0.002122 | -1.07974 |
| **CRYM** | 9.81E-08 | 0.002134 | -1.21725 |
| **SLPI** | 9.84E-08 | 0.002141 | -1.82892 |
| **TOM1** | 9.86E-08 | 0.002146 | -1.57301 |
| **EDN3** | 9.92E-08 | 0.002158 | -1.79928 |
| **RIOK3** | 1.05E-07 | 0.002278 | -1.08739 |
| **SLC16A7** | 1.06E-07 | 0.002303 | -1.78576 |
| **DSC2** | 1.11E-07 | 0.002404 | -1.81838 |
| **ACOX3** | 1.16E-07 | 0.002518 | -1.31324 |
| **PKP3** | 1.21E-07 | 0.002632 | -1.26985 |
| **ALDH3B2** | 1.22E-07 | 0.00266 | -1.1895 |
| **ELOVL4** | 1.24E-07 | 0.002703 | -1.71808 |
| **CPPED1** | 1.37E-07 | 0.00298 | -1.42567 |
| **FGFBP1** | 1.38E-07 | 0.003 | -1.17601 |
| **OCLN** | 1.39E-07 | 0.003015 | -1.34287 |
| **KRT78** | 1.41E-07 | 0.003078 | -2.99071 |
| **CYP2E1** | 1.42E-07 | 0.003094 | -1.42555 |
| **PHLDA1** | 1.43E-07 | 0.00311 | -1.34499 |
| **MFSD5** | 1.48E-07 | 0.003224 | -1.31987 |
| **CPEB3** | 1.50E-07 | 0.003274 | -1.3827 |
| **DBNDD1** | 1.51E-07 | 0.00329 | -1.00881 |
| **GULP1** | 1.52E-07 | 0.003307 | -1.36018 |
| **ALAD** | 1.57E-07 | 0.003409 | -1.03411 |
| **CRISP2** | 1.57E-07 | 0.003409 | -1.12295 |
| **HS3ST1** | 1.58E-07 | 0.003444 | -1.02709 |
| **UNC13B** | 1.58E-07 | 0.003444 | -1.23984 |
| **XK** | 1.59E-07 | 0.003469 | -1.48827 |
| **PRSS8** | 1.59E-07 | 0.003469 | -1.41501 |
| **CAB39L** | 1.73E-07 | 0.003769 | -1.56438 |
| **FNDC4** | 1.79E-07 | 0.003896 | -1.18038 |
| **ISOC1** | 1.81E-07 | 0.003942 | -1.12245 |
| **PRSS27** | 1.82E-07 | 0.003951 | -2.71236 |
| **ZNF750** | 1.83E-07 | 0.003978 | -1.67381 |
| **SUOX** | 1.86E-07 | 0.00404 | -1.00952 |
| **TM7SF2** | 1.87E-07 | 0.004062 | -1.56745 |
| **VPS4B** | 1.87E-07 | 0.004062 | -1.28297 |
| **ARHGAP10** | 2.03E-07 | 0.004411 | -1.08592 |
| **RNF141** | 2.06E-07 | 0.004476 | -1.3236 |
| **ACAA1** | 2.11E-07 | 0.004592 | -1.19502 |
| **CHMP2B** | 2.14E-07 | 0.004652 | -1.12138 |
| **TMEM80** | 2.15E-07 | 0.004674 | -1.03249 |
| **KRCC1** | 2.16E-07 | 0.004697 | -1.08188 |
| **ETHE1** | 2.26E-07 | 0.004927 | -1.18341 |
| **OXSR1** | 2.29E-07 | 0.004976 | -1.05902 |
| **VSIG10L** | 2.29E-07 | 0.004977 | -2.54348 |
| **TMEM9B** | 2.30E-07 | 0.004998 | -1.05694 |
| **PALMD** | 2.43E-07 | 0.00529 | -1.3739 |
| **CAMK2N1** | 2.50E-07 | 0.005437 | -1.51496 |
| **ARHGAP32** | 2.57E-07 | 0.005595 | -1.27846 |
| **PPFIBP2** | 2.68E-07 | 0.005834 | -1.13427 |
| **PLCD1** | 2.71E-07 | 0.005888 | -1.07443 |
| **ZDHHC13** | 2.73E-07 | 0.005932 | -1.49877 |
| **CCDC6** | 2.73E-07 | 0.005932 | -1.2597 |
| **FCGBP** | 2.89E-07 | 0.00628 | -1.26366 |
| **PDZRN3** | 2.90E-07 | 0.006309 | -1.1415 |
| **STK39** | 2.98E-07 | 0.006484 | -1.49652 |
| **DEPTOR** | 3.00E-07 | 0.006523 | -1.23884 |
| **OBFC1** | 3.03E-07 | 0.006585 | -1.696 |
| **KIAA0232** | 3.11E-07 | 0.006772 | -1.27523 |
| **ITPR2** | 3.15E-07 | 0.006846 | -1.15553 |
| **CLDN7** | 3.25E-07 | 0.007065 | -1.35528 |
| **ACADM** | 3.26E-07 | 0.007095 | -1.46096 |
| **KLF4** | 3.45E-07 | 0.007497 | -1.54483 |
| **FAM63A** | 3.58E-07 | 0.007791 | -1.13603 |
| **NAPA** | 3.61E-07 | 0.007847 | -1.31402 |
| **ALDH9A1** | 3.71E-07 | 0.008063 | -1.41413 |
| **PLD1** | 3.79E-07 | 0.008249 | -1.0623 |
| **EREG** | 3.89E-07 | 0.008468 | -2.19231 |
| **PLAGL1** | 3.91E-07 | 0.008504 | -1.36842 |
| **TFAP2B** | 3.95E-07 | 0.008585 | -1.77965 |
| **ZFP36** | 3.96E-07 | 0.008616 | -1.19371 |
| **LY6G6C** | 4.12E-07 | 0.008974 | -1.21199 |
| **FUT2** | 4.19E-07 | 0.009113 | -1.17621 |
| **CYP2C9** | 4.26E-07 | 0.009271 | -1.09714 |
| **SPINT1** | 4.26E-07 | 0.009271 | -1.02261 |
| **CD207** | 4.47E-07 | 0.009717 | -1.83121 |
| **CRYL1** | 4.49E-07 | 0.009758 | -1.42427 |
| **C12orf29** | 4.54E-07 | 0.009879 | -1.20726 |
| **LRRC20** | 4.76E-07 | 0.010353 | -1.09155 |
| **S100A14** | 4.98E-07 | 0.010842 | -1.54983 |
| **GAS7** | 5.03E-07 | 0.010933 | -1.08292 |
| **TRIM13** | 5.08E-07 | 0.011042 | -1.12779 |
| **PINK1** | 5.09E-07 | 0.01107 | -1.03764 |
| **GMDS** | 5.12E-07 | 0.011136 | -1.34994 |
| **LMO2** | 5.15E-07 | 0.011209 | -1.1158 |
| **DHRS11** | 5.30E-07 | 0.011539 | -1.02983 |
| **KLK6** | 5.37E-07 | 0.011682 | -1.8079 |
| **VAT1** | 5.38E-07 | 0.011712 | -1.13762 |
| **KLF8** | 5.41E-07 | 0.011779 | -1.39279 |
| **COBL** | 5.50E-07 | 0.011957 | -1.68129 |
| **EPHA2** | 5.57E-07 | 0.012123 | -1.37255 |
| **MAOA** | 5.66E-07 | 0.012312 | -1.09631 |
| **PIGN** | 5.66E-07 | 0.012312 | -1.15219 |
| **HERC6** | 5.97E-07 | 0.012992 | -1.04386 |
| **SORBS2** | 6.07E-07 | 0.013204 | -1.30883 |
| **OSTF1** | 6.09E-07 | 0.013258 | -1.14818 |
| **SLC12A6** | 6.14E-07 | 0.013365 | -1.07921 |
| **PDZK1IP1** | 6.21E-07 | 0.013508 | -1.63735 |
| **ELL2** | 6.29E-07 | 0.013692 | -1.05573 |
| **ADIRF** | 6.72E-07 | 0.014623 | -1.51164 |
| **HIGD1A** | 7.01E-07 | 0.015245 | -1.17799 |
| **TMEM57** | 7.10E-07 | 0.015443 | -1.15267 |
| **CD1A** | 7.15E-07 | 0.015548 | -1.48702 |
| **TRIM16** | 7.23E-07 | 0.015733 | -1.20288 |
| **ABHD5** | 7.35E-07 | 0.015981 | -1.15933 |
| **HMOX1** | 7.74E-07 | 0.016834 | -1.29625 |
| **RAB11FIP1** | 7.80E-07 | 0.016965 | -1.4777 |
| **ARHGEF10L** | 8.03E-07 | 0.017475 | -1.08685 |
| **ITPKC** | 8.23E-07 | 0.017907 | -1.01465 |
| **TGM5** | 8.36E-07 | 0.018184 | -1.42545 |
| **PPIC** | 8.51E-07 | 0.01851 | -1.08718 |
| **TIAM1** | 8.62E-07 | 0.018747 | -1.15269 |
| **ALDH3A1** | 8.95E-07 | 0.019471 | -1.53658 |
| **NBEAL2** | 9.04E-07 | 0.019671 | -1.0483 |
| **RAB5B** | 9.09E-07 | 0.019767 | -1.00698 |
| **ABCA8** | 9.26E-07 | 0.020147 | -1.79424 |
| **SH3GLB1** | 9.57E-07 | 0.020813 | -1.06734 |
| **TSPAN6** | 9.67E-07 | 0.021048 | -1.2949 |
| **AHNAK** | 1.01E-06 | 0.021928 | -1.39359 |
| **GRN** | 1.01E-06 | 0.021939 | -1.04067 |
| **CD59** | 1.02E-06 | 0.022256 | -1.01747 |
| **GJB5** | 1.04E-06 | 0.022588 | -1.02889 |
| **USO1** | 1.08E-06 | 0.023603 | -1.01827 |
| **PDCD6IP** | 1.13E-06 | 0.024649 | -1.09705 |
| **DUOX2** | 1.13E-06 | 0.024649 | -1.23689 |
| **PLS1** | 1.16E-06 | 0.025186 | -1.32419 |
| **GNAI3** | 1.18E-06 | 0.025578 | -1.02187 |
| **ARL6IP5** | 1.18E-06 | 0.02567 | -1.0316 |
| **FAM3D** | 1.20E-06 | 0.026197 | -2.58272 |
| **LOC101928830** | 1.25E-06 | 0.027235 | -1.10163 |
| **SPAG1** | 1.26E-06 | 0.027427 | -1.12391 |
| **CDK7** | 1.29E-06 | 0.027981 | -1.09534 |
| **5-Mar** | 1.29E-06 | 0.028012 | -1.0445 |
| **TLR3** | 1.32E-06 | 0.028686 | -1.28229 |
| **OCA2** | 1.34E-06 | 0.029199 | -1.10747 |
| **RBPMS** | 1.37E-06 | 0.029825 | -1.09111 |
| **BDKRB1** | 1.40E-06 | 0.030462 | -1.03593 |
| **SLC6A1** | 1.42E-06 | 0.030785 | -1.79713 |
| **S100A12** | 1.42E-06 | 0.030929 | -1.56576 |
| **TMEM254** | 1.47E-06 | 0.031992 | -1.20043 |
| **PLIN3** | 1.53E-06 | 0.033239 | -1.17551 |
| **PDZD2** | 1.53E-06 | 0.033278 | -1.35332 |
| **TST** | 1.53E-06 | 0.033354 | -1.07493 |
| **LY6D** | 1.55E-06 | 0.033731 | -1.21003 |
| **SPRR1A** | 1.60E-06 | 0.034884 | -1.9063 |
| **DENND2D** | 1.63E-06 | 0.035357 | -1.14299 |
| **ADH1B** | 1.67E-06 | 0.03627 | -1.90767 |
| **SLK** | 1.67E-06 | 0.036313 | -1.18343 |
| **CPA4** | 1.69E-06 | 0.036846 | -1.16355 |
| **RABGGTA** | 1.75E-06 | 0.03799 | -1.12363 |
| **STX12** | 1.77E-06 | 0.038508 | -1.0071 |
| **TMEM246** | 1.78E-06 | 0.038638 | -1.00976 |
| **SH3GL1** | 1.78E-06 | 0.038638 | -1.01993 |
| **HEBP2** | 1.87E-06 | 0.040774 | -1.04948 |
| **KANK1** | 1.90E-06 | 0.041323 | -1.3643 |
| **HPSE** | 1.97E-06 | 0.042768 | -1.26712 |
| **SUCLG2** | 1.97E-06 | 0.042865 | -1.0712 |
| **DPYD** | 1.98E-06 | 0.043151 | -1.2068 |
| **RBM47** | 2.13E-06 | 0.046245 | -1.23208 |
| **LEXM** | 2.15E-06 | 0.046679 | -2.03851 |
| **A2ML1** | 2.15E-06 | 0.046679 | -2.15164 |
| **SH3GLB2** | 2.22E-06 | 0.048251 | -1.26276 |
| **LOC441178** | 2.26E-06 | 0.049174 | -2.29472 |
| **PCSK5** | 2.28E-06 | 0.049522 | -1.0531 |

Note: Red, upregulated genes; Green, downregulated genes
