# Supplementary material for: Identification of four genes and biological characteristics of esophageal squamous cell carcinoma by integrated bioinformatics analysis
Source: Cancer Cell Int. 2021 Feb 18;21:123. doi: 10.1186/s12935-021-01814-1 (PMC7890804; doi:10.1186/s12935-021-01814-1)
Supplement: Supplementary file 2 — Additional file 2: Table S2. Go analysis for up and down-regulated DEGs, respectively. [file 12935_2021_1814_MOESM2_ESM.docx]

**Additional file 2: Table S2 Go analysis for up and down-regulated DEGs, respectively**

| Category | ID | Term | Genes | FDR value |
| --- | --- | --- | --- | --- |
| BP | GO:0051301 | cell division | KIF23, CKS1B, PRC1, NEK2, KNTC1, CEP55, SPC25, NCAPH, SAC3D1, NCAPG, CCNA1, ASPM, CDK1, CDC6, NUSAP1, CENPE, NDC80, CDC20, UBE2C, CDC25B, NCAPD2, SPAG5, BUB1B, RUVBL1, MYH10 | 1.30E-06 |
| CC | GO:0044420 | extracellular matrix part | COL4A2, COL4A1, LUM, TNC, COL3A1, SPARC, NID2, COL16A1, COL5A2, COL5A1, COL4A5, LAMB3, COL7A1, COL6A3, COL1A2, LAMC2, MFAP2, COL1A1, LOX, LAMB1, COL11A1, FN1, COL10A1 | 1.44E-13 |
| BP | GO:0000279 | M phase | KIF23, PRC1, NEK2, KNTC1, TTK, CEP55, SPC25, NCAPH, SAC3D1, NCAPG, CCNA1, ASPM, TRIP13, CDK1, CDC6, DLGAP5, TPX2, KIF18A, NUSAP1, CENPE, NDC80, CDC20, PBK, UBE2C, CDC25B, NCAPD2, SPAG5, BUB1B, RUVBL1 | 1.55E-08 |
| BP | GO:0032963 | collagen metabolic process | MMP10, TRAM2, MMP9, COL3A1, COL1A1, MMP3, SERPINH1, MMP13, COL5A1, MMP1 | 1.57E-06 |
| CC | GO:0005604 | basement membrane | LAMB3, COL4A2, COL4A1, COL7A1, TNC, LAMC2, NID2, SPARC, LAMB1, COL5A1, COL4A5, FN1 | 1.71E-04 |
| CC | GO:0005819 | spindle | KIF14, KIF23, CDC6, CDK1, KIF4A, PRC1, NEK2, DLGAP5, KNTC1, TPX2, KIF18A, NUSAP1, TTK, CDC20, CENPE, SAC3D1, SPAG5, BUB1B, ASPM, MYH10 | 1.96E-08 |
| MF | GO:0005201 | extracellular matrix structural constituent | COL4A2, COL4A1, BGN, LUM, COL3A1, COL1A2, COL1A1, FBN2, LAMB1, COL11A1, COL5A2, COL5A1, COL4A5, FN1 | 1.97E-06 |
| BP | GO:0022403 | cell cycle phase | KIF23, BCAT1, PRC1, BLM, NEK2, DBF4, KNTC1, TTK, CEP55, SPC25, NCAPH, SAC3D1, NCAPG, CCNA1, ASPM, TRIP13, CDK1, CDC6, DLGAP5, TPX2, KIF18A, NUSAP1, CENPE, NDC80, CDC20, PBK, CDKN3, UBE2C, NCAPD2, CDC25B, INHBA, SPAG5, BUB1B, RUVBL1, ACVR1 | 2.09E-10 |
| BP | GO:0044236 | multicellular organismal metabolic process | MMP10, TRAM2, MMP9, COL3A1, COL1A1, MMP3, SERPINH1, MMP13, COL5A1, MMP1 | 2.45E-05 |
| BP | GO:0000070 | mitotic sister chromatid segregation | NCAPH, NEK2, NCAPG, DLGAP5, KIF18A, NUSAP1, CENPE, NDC80, NCAPD2 | 3.41E-04 |
| CC | GO:0005583 | fibrillar collagen | LUM, COL3A1, COL1A2, COL1A1, COL5A2, COL11A1, COL5A1 | 3.45E-05 |
| BP | GO:0000280 | nuclear division | KIF23, NEK2, KNTC1, CEP55, SPC25, NCAPH, SAC3D1, NCAPG, CCNA1, ASPM, CDK1, CDC6, DLGAP5, KIF18A, TPX2, NUSAP1, CENPE, NDC80, CDC20, PBK, UBE2C, CDC25B, NCAPD2, SPAG5, BUB1B, RUVBL1 | 3.52E-10 |
| Category | **ID** | **Term** | **Genes** | **FDR value** |
| BP | GO:0008544 | epidermis development | KLK7, CRABP2, ANXA1, KRT13, GJB5, SPINK5, ALDH3A2, SCEL, BARX2, EVPL, EREG, SPRR2C, CST6, FLG, SPRR1A, PPL, SPRR2B, TGM1, SPRR3, TGM3, TGM5, IVL, EMP1, KLF4 | 3.72E-08 |
| BP | GO:0007398 | ectoderm development | KLK7, CRABP2, ANXA1, KRT13, GJB5, SPINK5, ALDH3A2, SCEL, BARX2, EVPL, EREG, SPRR2C, CST6, FLG, SPRR1A, PPL, SPRR2B, TGM1, SPRR3, TGM3, TGM5, IVL, EMP1, KLF4 | 1.88E-07 |
| BP | GO:0030855 | epithelial cell differentiation | ANXA1, DHRS9, EHF, SPINK5, SCEL, EVPL, RHCG, EREG, SPRR2C, SPRR1A, FLG, PPL, UPK1A, SPRR2B, TGM1, SPRR3, TGM3, KRT4, IVL, EMP1 | 3.55E-07 |
| CC | GO:0001533 | cornified envelope | EVPL, CST6, SPRR2C, FLG, SPRR1A, TGM1, SPRR2B, ANXA1, IVL, SCEL | 5.73E-07 |
| BP | GO:0009913 | epidermal cell differentiation | ANXA1, SPINK5, SCEL, EVPL, SPRR2C, EREG, FLG, SPRR1A, PPL, SPRR2B, TGM1, SPRR3, TGM3, IVL, KLF4 | 9.40E-07 |
| BP | GO:0006631 | fatty acid metabolic process | FCER1A, ACADM, CYP2J2, ABHD5, PTGS1, EPHX2, ECHDC2, ADH7, ACOX3, CRYL1, ALOX15B, ELOVL4, CH25H, ETFDH, SLC27A6, CYP4F3, ELOVL6, HPGD, MGST2, ACAA1, ALOX12 | 3.32E-05 |
| BP | GO:0030216 | keratinocyte differentiation | ANXA1, SCEL, EVPL, SPRR2C, EREG, SPRR1A, FLG, PPL, TGM1, SPRR2B, SPRR3, TGM3, IVL | 3.66E-05 |
| CC | GO:0005792 | microsome | KLK6, CYP3A5, PLD1, CYP2J2, CYP2C18, CYP2C9, PTGS1, DHRS9, CYP4F12, CYP2E1, ITPR2, CYP4B1, PPP1R3C, UGT1A3, SH3GLB1, HMOX1, FMO2, USO1, SORT1, CYP4F3, MGST2 | 4.06E-04 |
| CC | GO:0042598 | vesicular fraction | KLK6, CYP3A5, PLD1, CYP2J2, CYP2C18, CYP2C9, PTGS1, DHRS9, CYP4F12, CYP2E1, ITPR2, CYP4B1, PPP1R3C, UGT1A3, SH3GLB1, HMOX1, FMO2, USO1, SORT1, CYP4F3, MGST2 | 6.49E-04 |
| BP | GO:0060429 | epithelium development | ANXA1, DHRS9, EHF, SPINK5, SCEL, EVPL, RHCG, EREG, SPRR2C, SPRR1A, FLG, PPL, UPK1A, SPRR2B, TGM1, SPRR3, TGM3, KRT4, IVL, EMP1 | 0.001450374 |
| MF | GO:0046906 | tetrapyrrole binding | CYP3A5, SUOX, CYP2J2, CYP11A1, CYP2C18, CYP2C9, PTGS1, DUOX2, DUOX1, CYP4F12, CYP2E1, TCN1, CYP4B1, HMOX1, CYP4F3 | 0.00398058 |
| BP | GO:0031424 | keratinization | EVPL, SPRR2C, SPRR1A, PPL, TGM1, SPRR2B, SPRR3, TGM3, IVL | 0.007222866 |

Note: Red and Green represented Go analysis for up and down-regulated DEGs, respectively
