# Supplementary material for: Identification of four genes and biological characteristics of esophageal squamous cell carcinoma by integrated bioinformatics analysis
Source: Cancer Cell Int. 2021 Feb 18;21:123. doi: 10.1186/s12935-021-01814-1 (PMC7890804; doi:10.1186/s12935-021-01814-1)
Supplement: Supplementary file 3 — Additional file 3: Table S3. KEGG analysis for up and down-regulated DEGs, respectively. [file 12935_2021_1814_MOESM3_ESM.docx]

**Additional file 3: Table S3 KEGG analysis for up and down-regulated DEGs, respectively**

| Term | Count | Ratio | FDR |
| --- | --- | --- | --- |
| hsa04110:Cell cycle | 58 | 0.204002673 | 7.02E-10 |
| hsa03030:DNA replication | 26 | 0.091449474 | 1.81E-08 |
| hsa05222:Small cell lung cancer | 36 | 0.126622349 | 3.73E-04 |
| hsa03050:Proteasome | 23 | 0.080897612 | 0.006240547 |
| hsa03410:Base excision repair | 19 | 0.066828462 | 0.008693765 |
| hsa04512:ECM-receptor interaction | 32 | 0.112553199 | 0.034443915 |
| Term | **Count** | **Ratio** | **FDR** |
| hsa00982:Drug metabolism | 12 | 0.269905533 | 2.35E-04 |
| hsa00590:Arachidonic acid metabolism | 11 | 0.247413405 | 8.13E-04 |
| hsa00980:Metabolism of xenobiotics by cytochrome P450 | 10 | 0.224921278 | 0.013470948 |

Note: Red and Green represented KEGG analysis for up and down-regulated DEGs, respectively
